# Cellular heterogeneity of pluripotent stem cell-derived cardiomyocyte grafts is mechanistically linked to treatable arrhythmias

**DOI:** 10.1038/s44161-023-00419-3

**Published:** 2024-02-06

**Authors:** Dinesh Selvakumar, Zoe E. Clayton, Andrew Prowse, Steve Dingwall, Sul Ki Kim, Leila Reyes, Jacob George, Haisam Shah, Siqi Chen, Halina H. L. Leung, Robert D. Hume, Laurentius Tjahjadi, Sindhu Igoor, Rhys J. P. Skelton, Alfred Hing, Hugh Paterson, Sheryl L. Foster, Lachlan Pearson, Emma Wilkie, Alan D. Marcus, Prajith Jeyaprakash, Zhixuan Wu, Han Shen Chiu, Cherica Felize J. Ongtengco, Onkar Mulay, Jeffrey R. McArthur, Tony Barry, Juntang Lu, Vu Tran, Richard Bennett, Yasuhito Kotake, Timothy Campbell, Samual Turnbull, Anunay Gupta, Quan Nguyen, Guiyan Ni, Stuart M. Grieve, Nathan J. Palpant, Faraz Pathan, Eddy Kizana, Saurabh Kumar, Peter P. Gray, James J. H. Chong

**Affiliations:** 1 Centre for Heart Research, the Westmead Institute for Medical Research, the University of Sydney, Westmead, New South Wales Australia; 2 Department of Cardiology, Westmead Hospital, Westmead, New South Wales Australia; 3 Australian Institute for Bioengineering and Nanotechnology, the University of Queensland, St Lucia, Queensland Australia; 4 Department of Cardiothoracic Surgery, Westmead Hospital, Westmead, New South Wales Australia; 5 Sydney Imaging, Core Research Facility, the University of Sydney, Sydney, New South Wales Australia; 6 Department of Radiology, Westmead Hospital, Westmead, New South Wales Australia; 7 Sydney School of Health Sciences, Faculty of Medicine and Health, the University of Sydney, Sydney, New South Wales Australia; 8 Department of Cardiology, Nepean Hospital, Kingswood, New South Wales Australia; 9 Institute for Molecular Bioscience, the University of Queensland, St Lucia, Queensland Australia; 10 Genomics and Machine Learning Lab, Division of Genetics and Genomics, Institute for Molecular Bioscience, the University of Queensland, St Lucia, Queensland Australia; 11 Victor Chang Cardiac Research Institute, Darlinghurst, New South Wales Australia; 12 St. Vincent’s Clinical School, UNSW, Darlinghurst, New South Wales Australia; 13 Imaging and Phenotyping Laboratory, Faculty of Medicine and Health, Charles Perkins Centre, the University of Sydney, Sydney, New South Wales Australia; 14 Sydney Medical School, Charles Perkins Centre Nepean, Faculty of Medicine and Health, the University of Sydney, Sydney, New South Wales Australia

**Keywords:** Cardiac regeneration, Stem-cell research

## Abstract

Preclinical data have confirmed that human pluripotent stem cell-derived cardiomyocytes (PSC-CMs) can remuscularize the injured or diseased heart, with several clinical trials now in planning or recruitment stages. However, because ventricular arrhythmias represent a complication following engraftment of intramyocardially injected PSC-CMs, it is necessary to provide treatment strategies to control or prevent engraftment arrhythmias (EAs). Here, we show in a porcine model of myocardial infarction and PSC-CM transplantation that EAs are mechanistically linked to cellular heterogeneity in the input PSC-CM and resultant graft. Specifically, we identify atrial and pacemaker-like cardiomyocytes as culprit arrhythmogenic subpopulations. Two unique surface marker signatures, signal regulatory protein α (SIRPA)^+^CD90^−^CD200^+^ and SIRPA^+^CD90^−^CD200^−^, identify arrhythmogenic and non-arrhythmogenic cardiomyocytes, respectively. Our data suggest that modifications to current PSC-CM-production and/or PSC-CM-selection protocols could potentially prevent EAs. We further show that pharmacologic and interventional anti-arrhythmic strategies can control and potentially abolish these arrhythmias.

## Abstract (web summary)

Selvakumar, Clayton et al. use a porcine model of myocardial infarction and PSC-CM transplantation and identify atrial and pacemaker-like cardiomyocytes as the cause of engraftment arrhythmias and surface marker signatures to distinguish between arrhythmogenic and non-arrhythmogenic cardiomyocytes.

## Main

Myocardial infarction (MI), the leading cause of heart failure, results in the loss of up to 1 billion highly specialized cardiomyocytes (CMs)^1^. Despite great interest, numerous attempts to replace damaged or destroyed CMs with cell therapy have yielded inconsistent results in clinical trials^2–4^. This may be partly attributable to the lack of cardiomyogenic differentiation capacity of the tested cell types^5,6^. Pluripotent stem cells, however, are a renewable source of CMs^7^. Exciting preclinical data have confirmed that PSC-CMs can remuscularize and improve cardiac function in clinically relevant large-animal models^8–13^. Several clinical trials are now in planning or recruitment stages worldwide^14–18^. However, hurdles to widescale clinical translation remain^19,20^. The most concerning of these relates to ventricular arrhythmias, which ensue following intramyocardial PSC-CM delivery, hereafter referred to as EAs^8–12^.

There is a paucity of literature examining the mechanism of EA, with existing evidence suggesting that arrhythmias arise due to the abnormal impulse generation and enhanced automaticity of PSC-CM grafts^9,11^. Furthermore, there has only been one study on therapeutic strategies to mitigate EAs^12^, with this recent report suggesting that pharmacotherapy can suppress but not abolish arrhythmias. Therefore, as PSC-CMs enter the clinical arena, there is an urgent need to gain further mechanistic insights and test additional means of therapeutic arrhythmia control.

Current PSC-CM differentiation protocols yield heterogeneous cellular outputs. Although ventricular CMs predominate, other cell types such as atrial and pacemaker-like cells are also present^21–23^. Despite suggestions that purified ventricular CMs may be the most desirable cell product for transplantation applications^24^, to date no study has investigated the important relationship between cellular heterogeneity and arrhythmogenesis.

Here, we use a porcine MI model to study EAs after phenotyping the composition of PSC-CM cell doses. We aimed to identify cellular characteristics predictive of arrhythmogenesis, hypothesizing that this would inform the safe production of CMs for future clinical use. We also sought to test the efficacy of clinically available pharmacologic and procedural anti-arrhythmic treatments that could abrogate EAs should they arise in clinical trials.

## Results

### Bioreactor production of PSC-CMs

**Extended Data Fig. 1:**
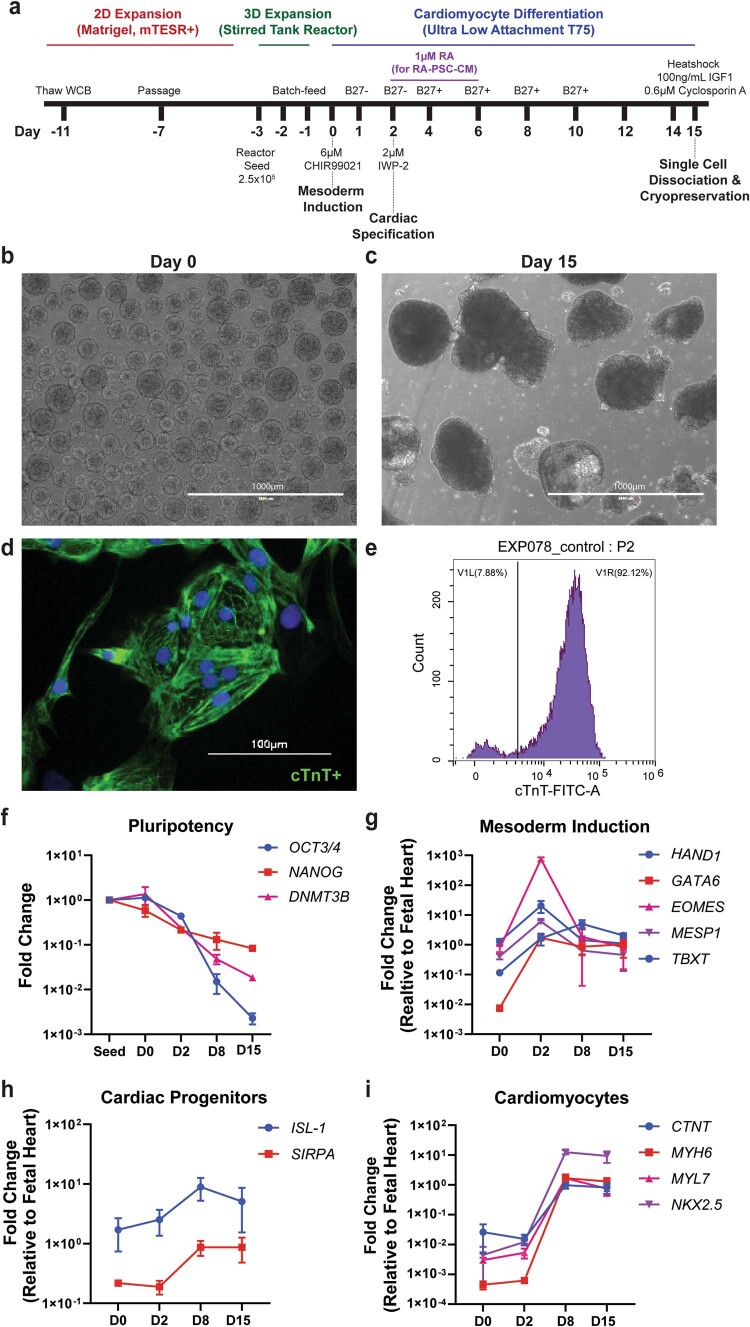
Stirred tank bioreactor production of PSC-CMs for porcine transplantation experiments. **(a)** Schematic depicting PSC-CM stirred tank reactor production protocol. **(b)** Appearance of cells at day 0 and **(c)** day 15 of differentiation protocol. **(d)** Immunostaining for cardiac troponin T demonstrating evidence of cytoskeletal formation in PSC-CMs. **(e)** Representative flow cytometric evaluation of PSC-CMs quantifying cardiac troponin T expression. **(f)** qPCR data showing expression profile of pluripotency, **(g)** mesodermal, **(h)** cardiac progenitor, and **(i)** cardiomyocyte markers over time course of differentiation protocol. All data in panels (f-i) is presented as mean ± SEM. n = 5 biologically independent experiments for each marker. Source data

Each batch of human CMs was generated from the same working cell bank material. CMs expressed cardiac troponin T (CTNT) and had evidence of cytoskeletal formation (Extended Data Fig. 1d,e). Genes for pluripotency, early mesoderm induction and cardiac specification were assessed via quantitative polymerase chain reaction (qPCR) over the time course of differentiation (Extended Data Fig. 1f–i). These genes exhibited expression trends comparable to those in previously published PSC-CM-production methods^25^.

### PSC-CM EAs are focal and automatic

After the generation of sufficient PSC-CMs, we conducted transplantation experiments over three phases in 31 landrace swine. Of these, two subjects died following induction of MI, one died due to procedural complications following thoracotomy, and another died a week following cell injection due to severe sepsis and infective endocarditis.

**Fig. 1:**
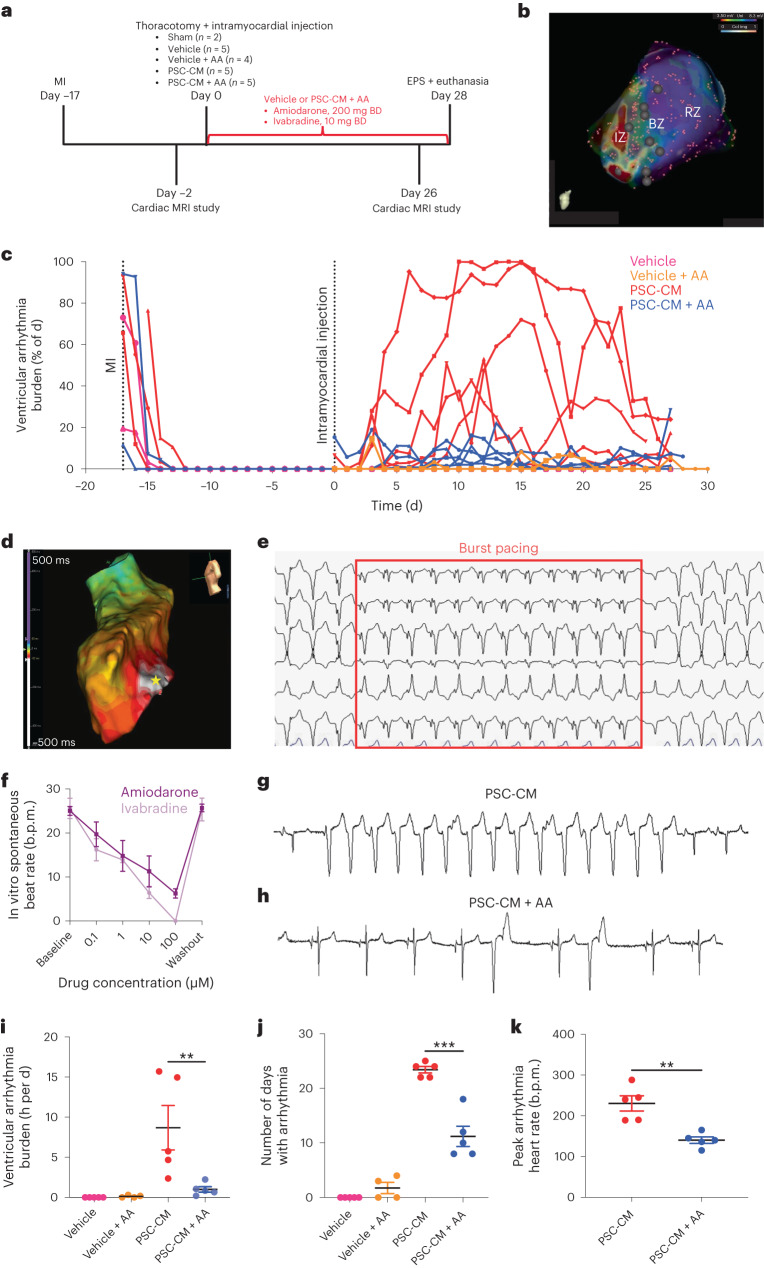
Focal, automatic EAs can be effectively suppressed with pharmacotherapy. **a**, Study timeline for phase 1 large-animal experiments in which 19 swine were randomized to four treatment groups (vehicle, vehicle + AA, PSC-CM, PSC-CM + AA) following percutaneously induced MI. A further two animals underwent sham infarction and vehicle injection. BD, twice a day; EPS, electrophysiological study. **b**, Representative CMR reconstruction (created with ADAS 3D) merged with an epicardial voltage map, used to choose and annotate border and infarct zone injection sites (black circles). BZ, border zone; col, color; img, image; IZ, infarct zone; RZ, remote zone; Uni, unipolar voltage. **c**, Plot depicting the percentage of time per day that each cell (red), cell and drug (blue) or vehicle (pink) recipient spent in ventricular arrhythmia over the course of the study protocol. **d**, Representative endocardial activation map during EA localizing arrhythmia origin to focal sites of cell injections (white, early activation; purple, late activation; star, site of earliest activation). **e**, Representative EA persisting through burst pacing, suggesting an automatic mechanism rather than a re-entrant mechanism. **f**, Dose-dependent suppression of PSC-CM contraction rate using amiodarone (dark purple) and ivabradine (light purple) in vitro (drug concentrations of 0–100 µM, *n* = 3 biologically independent experiments for each group, presented as mean ± s.e.m.). **g**, Telemetry strip from a representative cell recipient exhibiting a run of EA with spontaneous onset and offset. **h**, Telemetry strip from representative cell and anti-arrhythmic recipients showing a predominantly sinus rhythm with occasional ventricular ectopic beats. **i**–**k**, Arrhythmia parameters from day 0 to day 28 for each subject, grouped by treatment allocation (vehicle, *n* = 5; vehicle + AA, *n* = 4; PSC-CM, *n* = 5; PSC-CM + AA, *n* = 5; biologically independent animals; data are presented as mean ± s.e.m.). Significant reduction in arrhythmia burden (**i**) (**P = 0.008, Mann–Whitney test, two-tailed), number of days with arrhythmia (**j**) (***P = 0.0002, unpaired *t*-test, degrees of freedom (df) = 8, two-tailed) and peak arrhythmia heart rate (**k**) (**P = 0.002, unpaired *t*-test, df = 8, two-tailed) in cell recipients treated with anti-arrhythmics. Source data

**Extended Data Fig. 2:**
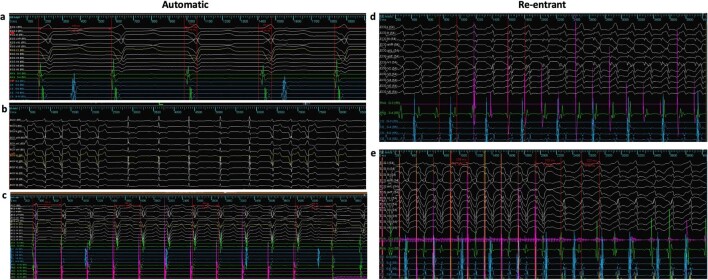
Electrocardiograms demonstrating automatic and re-entrant mechanisms for ventricular arrhythmias from representative cell and vehicle recipients. **(a-c)** Electrograms from spontaneous ventricular tachycardia in cell recipient showing characteristics consistent with automatic arrhythmia mechanism. **(a)** Electrocardiograms from site of earliest activation during spontaneous ventricular tachycardia in cell recipient. Corresponding activation map viewable in Fig. 1d. Electrogram duration=73 ms which is 18% of the ventricular tachycardia cycle length (410 ms). **(b)** Transient suppression and spontaneous re-initiation of ventricular tachycardia in cell recipient. **(c)** Instant capture during left ventricular pacing, followed by instant continuation of ventricular tachycardia. Post-pacing interval – tachycardia cycle length ~ 114 ms. **(d-e)** Electrograms from induced ventricular arrhythmia in vehicle recipients showing characteristics consistent with re-entrant arrhythmia mechanism. **(d)** Initial fusion evident at onset of right ventricular overdrive pacing during induced ventricular tachycardia. **(e)** Clean resumption of tachycardia after overdriving pacing. Post-pacing interval – tachycardia cycle length ~ 90 ms.

The first phase of our large-animal experiments was designed to gain insights into the electrophysiological nature of PSC-CM-related EAs and to assess the treatment efficacy of clinically available anti-arrhythmic agents (AA). Transplantation studies were completed in 21 subjects 2 weeks following percutaneously induced ischemia–reperfusion MI (Fig. 1a). Animals were randomized into one of five treatment groups: PSC-CM (*n* = 5), PSC-CM + AA (*n* = 5), vehicle (*n* = 5), vehicle + AA (*n* = 4) or sham MI with vehicle injection (*n* = 2). Seven hundred and fifty million PSC-CMs or vehicle were delivered into infarct and border zones via transepicardial injections following lateral thoracotomy. To enable accurate targeting and annotation of cell injections, we developed a technique in which epicardial voltage maps were merged with reconstructed cardiac magnetic resonance (CMR) generated from ADAS 3D imaging software (Fig. 1b). No spontaneous arrhythmias were observed in any vehicle-treated subjects; however, all cell-treated subjects developed EA within a week of PSC-CM transplantation (Fig. 1c). Follow-up electroanatomic mapping studies performed 4 weeks after transplantation localized EA origin to focal sites of cell injection (Fig. 1d and Extended Data Fig. 2a). Subsequent histological analyses confirmed these to be sites of cell engraftment.

In the same terminal mapping procedure, the EA mechanism was elucidated (Supplementary Table 1). All EAs occurred either spontaneously or after administration of the catecholaminergic drug isoprenaline. None occurred after programmed electrical stimulation (PES). Termination was spontaneous, typically followed by spontaneous reinitiation (Extended Data Fig. 2b). Sinus rhythm could not be restored by either rapid ventricular pacing (Fig. 1e) or external cardioversion. Cycle length variation was noted without changes in electrocardiogram (ECG) morphology. Attempts at entrainment accelerated the arrhythmia, without instances of constant or progressive fusion or tachycardia reset (Extended Data Fig. 2c). Together, these findings indicate enhanced automaticity as an EA mechanism.

By contrast, vehicle-treated animals only experienced arrhythmias that could be induced by rapid ventricular pacing, had fixed cycle lengths and could be terminated by rapid pacing or external cardioversion. These arrhythmias could be entrained from multiple right ventricular sites, leading to constant or progressive fusion along with tachycardia reset and continuation following the final entrained pacing beat (Extended Data Fig. 2d,e). This suggests scar-mediated re-entrant circuits as the mechanism for induced arrhythmias in vehicle-treated animals^26^, the typical cause of post-MI ventricular arrhythmia^27^.

### Pharmacological suppression of PSC-CM EAs

Given the enhanced automaticity mechanism for PSC-CM EAs, we hypothesized that suppressing the rate of graft automaticity below that of the native sinus node would reduce arrhythmia burden. Ivabradine, a selective inhibitor of the pacemaker current (I_f_) responsible for CM automaticity, and amiodarone, a widely used anti-arrhythmic drug that blocks multiple ion channels, were selected as the anti-arrhythmic drugs. In vitro experiments confirmed that both these drugs induced a dose-dependent reduction in the spontaneous beat rate of PSC-CMs cultured in monolayer (Fig. 1f). In vivo arrhythmia detection was performed through blinded analysis of continuous ECG data transmitted from implanted telemetry units. Although all animals experienced transient arrhythmias attributable to reperfusion injury immediately following MI, the sinus rhythm was maintained for several days leading up to epicardial injections (Fig. 1c). Amiodarone–ivabradine treatment was effective in suppressing EAs, with significant reduction of all telemetry parameters observed in drug-treated pigs (Fig. 1g–k and Supplementary Table 1): PSC-CM versus PSC-CM + AA, hours per day of arrhythmia (8.7 ± 2.8 h versus 0.99 ± 0.34 h; *P* < 0.05), days with arrhythmia (23.4 ± 0.6 d versus 11.2 ± 1.8 d; *P* < 0.0005) and peak arrhythmia heart rate (230.4 ± 18.7 beats per minute (b.p.m.) versus 140.2 ± 8.0 b.p.m.; *P* < 0.005).

### PSC-CMs with anti-arrhythmic therapy improve cardiac function

**Fig. 2:**
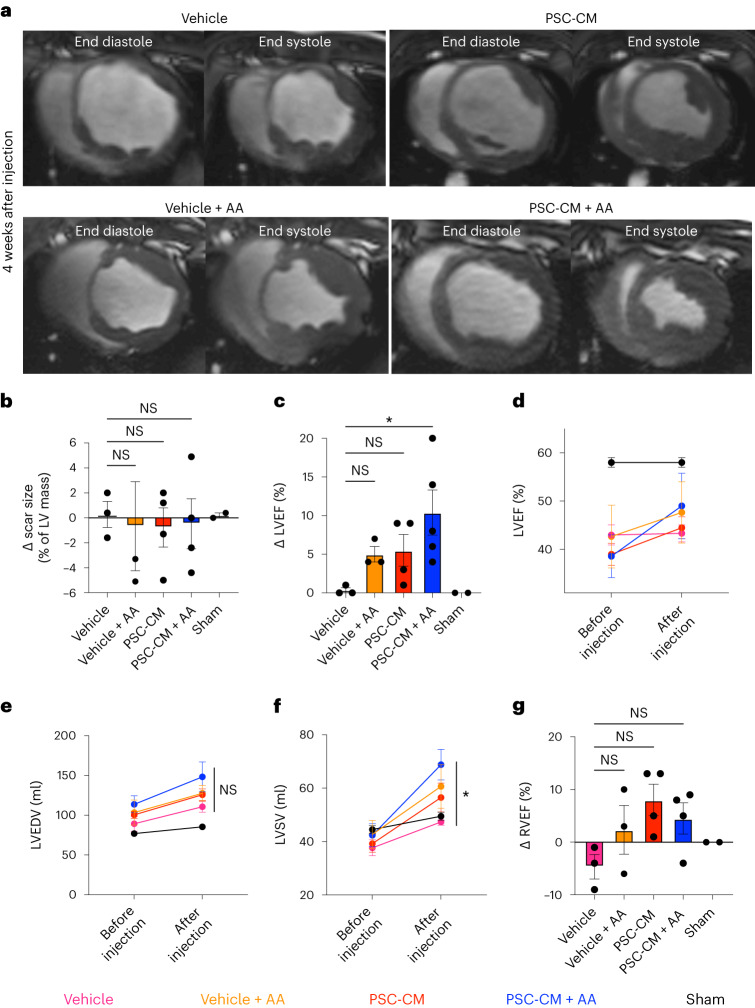
Effects of PSC-CM transplantation on cardiac volumes and function. **a**, Representative short-axis cine magnetic resonance imaging (MRI) from end-diastolic and end-systolic phases of the cardiac cycle 4 weeks after cell or vehicle injection. Greater ejection of blood in systole was observed for cell recipients than for vehicle recipients, most notably in cell recipients treated with anti-arrhythmic drugs. **b**, Plot depicting the change in (Δ) scar size from baseline (after MI, before injection) to 4 weeks after injection, with no significant difference between treatment groups (*P* = 0.93, Kruskal–Wallis test). **c**, Plot depicting the change in LVEF from baseline (after MI, before injection) to 4 weeks after injection, with significantly improved LVEF in cell and anti-arrhythmic recipients (vehicle versus vehicle + AA, *P* = 0.32; vehicle versus PSC-CM, *P* = 0.20; vehicle versus PSC-CM + AA, *P = 0.02; Kruskal–Wallis with Dunn’s multiple-comparison test). **d**–**g**, Pooled group data of left and right ventricular function at baseline (after MI, before injection) and 4 weeks after injection. The greatest improvement in LVEF (**d**) was noted in cell and anti-arrhythmic recipients. No significant change in LVEDV (**e**) was observed between groups (*P* = 0.76, Kruskal–Wallis test) although there was a significant improvement in LVSV (**f**) in cell and anti-arrhythmic recipients (vehicle versus vehicle + AA, *P* > 0.99; vehicle versus PSC-CM, *P* = 0.60; vehicle versus PSC-CM + AA, *P = 0.04; Kruskal–Wallis with Dunn’s multiple-comparison test). For RVEF (**g**), there was no significant difference between groups (*P* = 0.15, Kruskal–Wallis test). All data in **b**–**g** are presented as mean ± s.e.m. Experiments were conducted in biologically independent animals; vehicle, *n* = 3; PSC-CM, *n* = 3; PSC-CM + AA, *n* = 4; sham, *n* = 2. Source data

Although the primary goal of this study was not to demonstrate salutary effects with PSC-CM therapy, we nonetheless assessed cardiac structure and function with serial CMR. All subjects underwent CMR 2 d before and 4 weeks after transepicardial PSC-CM injections (Fig. 2a). Image analysis was conducted according to standard reporting guidelines by two blinded observers^28^. Four animals were excluded from functional analysis due to failure of infarct creation, all with scar sizes under 1.5% of left ventricular mass (Supplementary Table 2). Left ventricular ejection fraction (LVEF) was preserved in sham subjects (58% ± 1%) and similarly depressed in all infarcted animals before transepicardial injection (vehicle, 43% ± 2%; vehicle + AA, 43% ± 5%; PSC-CM, 39% ± 2%; PSC-CM + AA, 39% ± 4%; *P* = 0.44) (Supplementary Table 2). At the 4-week follow-up, no significant change in scar size, expressed as a percentage of total left ventricular mass, was demonstrated between groups (change in scar size: vehicle, 2% ± 1%; vehicle + AA, 0.7% ± 4%; PSC-CM, −0.8% ± 3%; PSC-CM + AA, −0.5% ± 2%; *P* = 0.93, not significant (NS)) (Fig. 2b). Despite this, a statistically significant improvement in LVEF was observed (change in LVEF: vehicle, 0.3% ± 0.3%; vehicle + AA, 5% ± 2%; PSC-CM, 6% ± 4%; PSC-CM + AA, 10% ± 3%; *P* < 0.05), with post hoc analysis showing that this was driven by the PSC-CM + AA group (Fig. 2c,d). To further interrogate this finding, we evaluated the impact of our interventions on left ventricular volumes. Despite improvement in stroke volume (LVSV) in PSC-CM + AA-treated subjects (change in LVSV: vehicle, 10 ± 2 ml; vehicle + AA; 11 ± 4 ml, PSC-CM, 17 ± 4 ml; PSC-CM + AA, 26 ± 4 ml; *P* < 0.05), changes in left ventricular end-diastolic volumes (LVEDVs) were comparable between groups and, if anything, greater in animals in the PSC-CM + AA group (change in LVEDV: vehicle, 21 ± 4 ml; vehicle + AA, 24 ± 8 ml; PSC-CM, 25 ± 5 ml; PSC-CM + AA, 34 ± 10 ml; *P* = 0.76, NS) (Fig. 2e,f). Together, these data suggest that the improvement in function observed in PSC-CM recipients may be attributable to greater left ventricular contractility rather than reduction of adverse remodeling and left ventricular dilation after MI, with the greatest benefit evident in animals with arrhythmias suppressed by drug therapy.

Therapeutic efficacy on right ventricular function was also assessed (Fig. 2g). Although a trend of improvement was evident in all cell recipients, this difference approached but did not reach statistical significance (change in right ventricular ejection fraction (RVEF): vehicle, −2% ± 4%; vehicle + AA, 2% ± 5%; PSC-CM, 8% ± 3%; PSC-CM ± AA, 5% ± 2%; *P* = 0.15, NS).

### PSC-CMs are heterogeneous with arrhythmogenic subpopulations

**Fig. 3:**
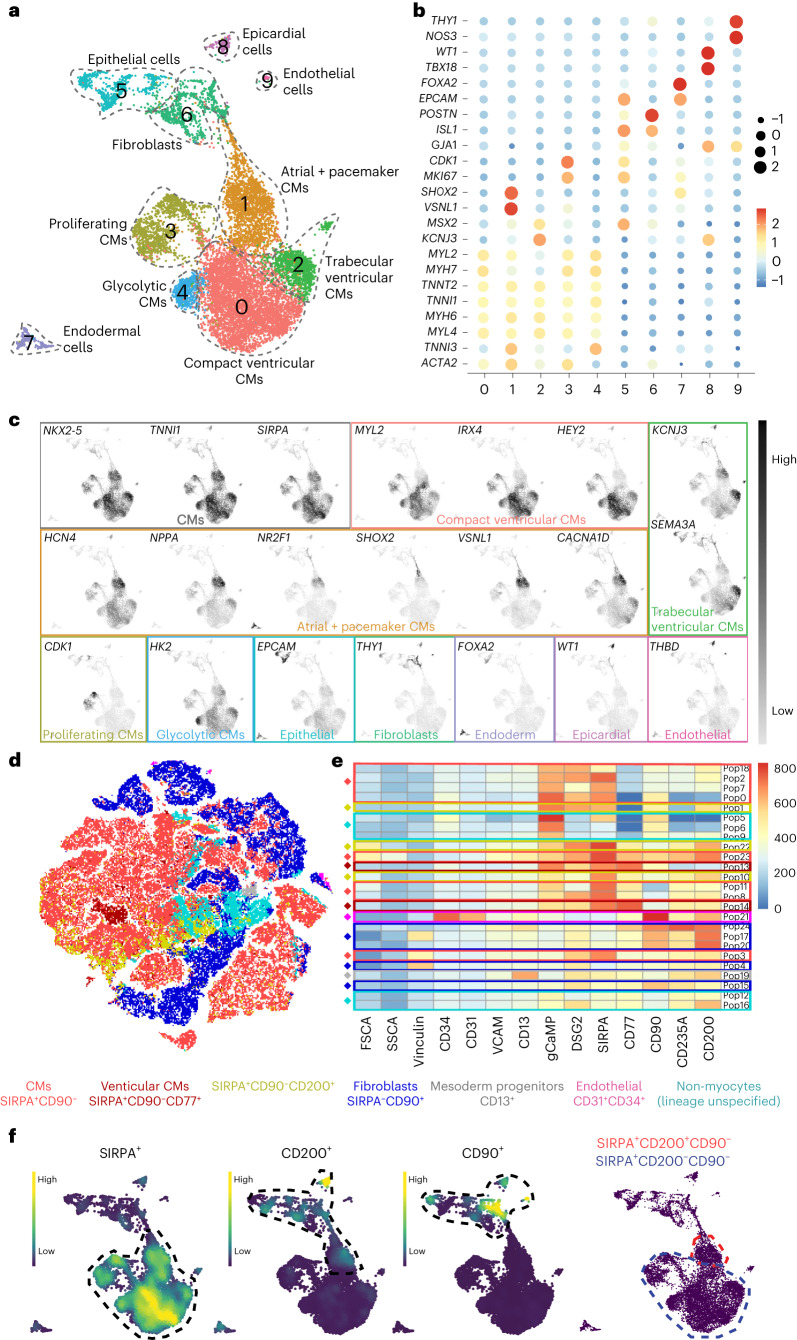
PSC-CM cell doses are heterogeneous with arrhythmogenic subpopulations. **a**, UMAP embedding of scRNA-seq data from representative samples of cell doses. Ten clusters were identified and annotated based on differential gene expression. **b**, Dot plot showing expression of representative marker genes used to categorize clusters. **c**, Nebulosa plots showing the density of specific marker gene expression based on gene-weighted density estimation, demarcating cell type identity to clusters. **d**, Representative *t*-SNE plot from a sample of a cell dose analyzed by flow cytometry. FlowSOM meta-clusters overlaid onto the *t*-SNE plot highlight several discrete subpopulations within the cell fraction, which were identified based on surface marker signatures. **e**, Heatmap showing relative expression of cell surface markers within each meta-cluster. DSG2, desmoglein 2; FSCA, forward scatter A; pop, population; SSCA, side scatter A; VCAM, vascular cell adhesion molecule. **f**, Nebulosa plots displaying expression levels of SIRPA, CD200 and CD90. The rightmost panel outlines SIRPA^+^CD90^−^CD200^−^ CMs, which were negatively associated with arrhythmias, and SIRPA^+^CD90^−^CD200^+^ CMs, which were positively associated with arrhythmias. SIRPA^+^CD90^−^CD200^+^ CMs comprise atrial and pacemaker CMs (cluster 1), and SIRPA^+^CD90^−^CD200^−^ CMs encompass all remaining CM clusters (clusters 0, 2, 3 and 4).

**Extended Data Fig. 3:**
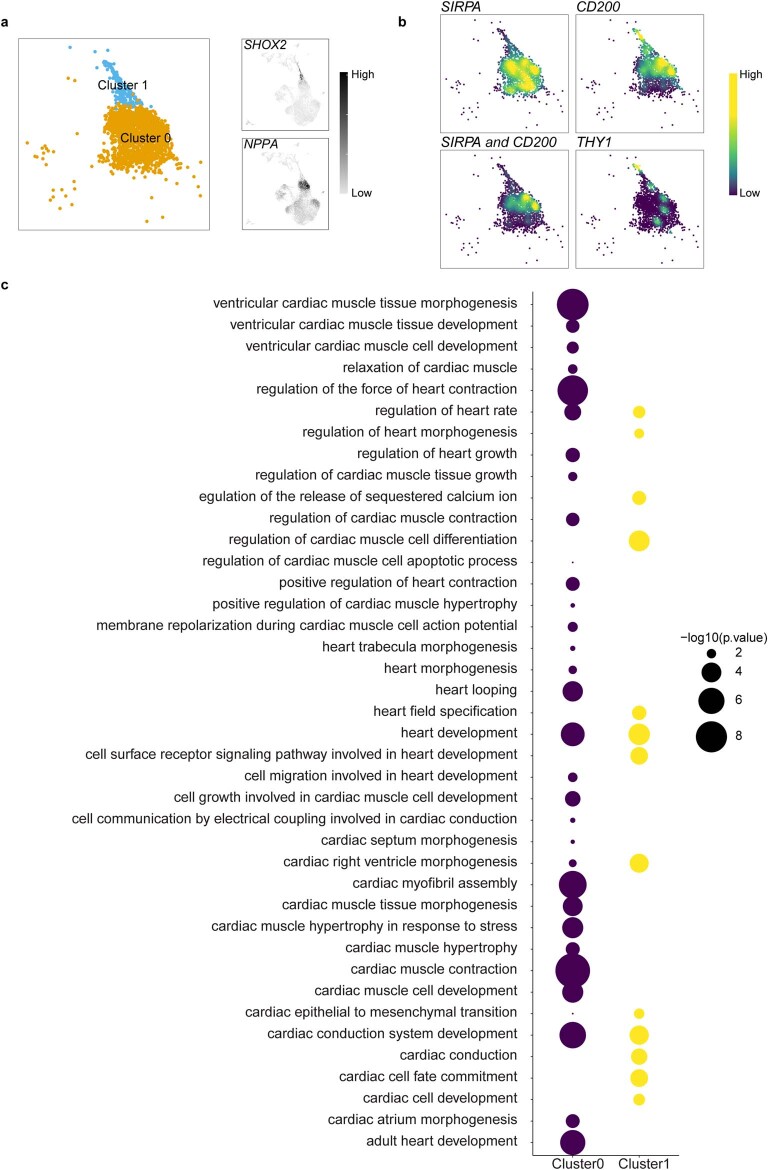
Subdivision of cardiomyocyte cluster 1 into atrial-like and pacemaker-like clusters. **(a)** UMAP plot showing subdivision of cluster 1 into atrial (0, orange) and pacemaker (1, blue) subclusters, with expression of cluster specific genes, *NPPA* and *SHOX2*, inset. **(b)** Relative expression of SIRPA, CD200, and CD90 in cluster 1, indicating the presence of SIRPA + /CD200 + /CD90^−^ arrhythmogenic PSC-CMs predominantly in the atrial sub-cluster. **(c)** List of GO terms for differentially expressed genes between atrial and pacemaker subclusters. The Fisher’s exact test was used to determine statistical significance.

We next sought to identify arrhythmogenic cellular characteristics that could be strategically targeted. Representative samples from each PSC-CM cell dose were retained before transplantation for use in single-cell RNA sequencing (scRNA-seq) and high-dimensional flow cytometry experiments. Uniform manifold approximation and projection (UMAP) plots of clustered scRNA-seq data identified ten distinct cellular subpopulations, the identities of which were surmised based on differential gene expression (Fig. 3a–c). Most cells expressed markers of a committed cardiac lineage such as *NKX2-5*, *SIRPA* and *TNNI1*, representing clusters 0–4. Within these CM populations, further heterogeneity was noted. Compact ventricular markers^29–31^ such as *MYL2*, *IRX4*, *MYH7* and *HEY2* were abundantly expressed in cluster 0, whereas atrial and pacemaker markers^32–34^ including *SHOX2*, *VSNL1*, *NPPA* and *NR2F1* were localized to cluster 1. Cluster 1 was then further subdivided into distinct atrial and pacemaker subclusters based on differential expression of cardiac contractile and conduction system-associated genes (Extended Data Fig. 3).

Markers of the trabecular myocardium such as *KCNJ3*, *SEMA3A*, *IRX3* and *SCN5A*^29,35^ were predominantly expressed in cluster 2, with proliferative markers^36^ such as *CDK1* and *MKI67* identifying cluster 3. Cluster 4 showed strong expression of the glycolytic marker *HK2* (ref. ^37^), demarcating an earlier-stage glycolytic CM population. Non-CM cell populations were also present, with clusters 5–9 inclusive of fibroblasts and epithelial, endodermal, epicardial and endothelial cells. Together, these data confirm the dynamic transcriptional heterogeneity of the profiled cells.

Characterization by way of high-parameter flow cytometry also confirmed heterogeneity (Fig. 3d,e, Extended Data Fig. 10). We designed a panel comprising 12 markers (ten cell surface markers, endogenous genetically encoded, intracellular calcium transient marker (gCaMP) and vinculin) to interrogate cell composition of the PSC-CM doses (Supplementary Table 3). Resultant *t*-distributed stochastic neighbor-embedding (*t*-SNE) plots overlaid with 25 FlowSOM meta-clusters were annotated when possible based on previously reported surface marker signatures. Of note, CMs were classified as SIRPA^+^CD90^−^ (ref. ^38^). Non-myocyte subpopulations were also present, with fibroblasts defined as SIRPA^−^CD90^+^ (refs. ^39,40^) and endothelial cells defined as CD34^+^CD31^+^ (ref. ^41^). A small subpopulation of CD13^+^ non-myocytes was identified, which may represent mesodermal progenitors^42^. Due to the limitations of the surface marker panel in determining cellular fate, several subpopulations could not be definitively labeled and were annotated as lineage-unspecified cells. Results of scRNA-seq (Fig. 3a) indicate that these populations likely represent epithelial or endodermal cells.

**Extended Data Fig. 4:**
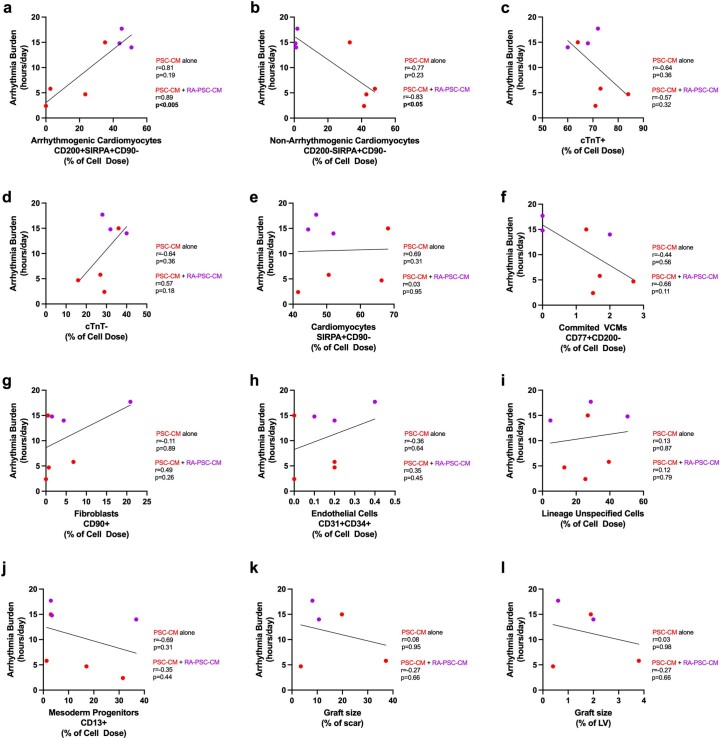
Correlation scatter plots demonstrating strength of linear association between flow cytometry derived subpopulation quantification or histologically determined graft size and resultant arrhythmia burden for each PSC-CM (red) and RA-PSC-CM (purple) cell dose. **(a)** Arrhythmogenic cardiomyocytes, SIRPA^+^CD90^−^CD200^+^ **(b)** Non-arrhythmogenic cardiomyocytes, SIRPA^+^CD90^−^CD200^−^ **(c)** Cardiac troponin T positive cells **(d)** Cardiac troponin T negative cells **(e)** Cardiomyocytes, SIRPA^+^CD90^−^ **(f)** Committed ventricular cardiomyocytes, CD77^+^CD200^−^ **(g)** Fibroblasts, CD90^+^ **(h)** Endothelial cells, CD31^+^CD34^+^ **(i)** Non-myocytes **(j)** Mesodermal progenitors, CD13^+^ **(k)** Graft size as percentage of infarct area in stained section **(l)** Graft size as percentage of left ventricular area in stained section. Pearson correlation reported in all panels. Source data

To compare the relative abundance of specific subpopulations within and between cell doses, data from all doses were concatenated and analyzed. This allowed subpopulation quantification, which was then correlated with total arrhythmia burden for each cell recipient (Table 1 and Extended Data Fig. 4). Subjects in the PSC-CM + AA group were excluded from this analysis due to the confounding effect of arrhythmia suppression. Interestingly, the strongest correlation between subpopulation quantification and arrhythmia burden occurred with a previously undefined SIRPA^+^CD90^−^CD200^+^ population (*r* = 0.81). Conversely, SIRPA^+^CD90^−^CD200^−^ cells had a negative association with arrhythmia burden (*r* = −0.77). To further define this surface marker signature, scRNA-seq data were interrogated. SIRPA^+^CD90^−^CD200^+^ cells comprise cluster 1 (atrial and pacemaker CMs), and SIRPA^+^CD90^−^CD200^−^ cells comprise all remaining CM clusters (Fig. 3f). In sum, these data confirm the cellular heterogeneity of transplanted PSC-CMs and identify a possible causal link between atrial and pacemaker-like CMs and arrhythmogenesis in PSC-CM-treated subjects.

**Table 1:** Arrhythmia burden and subpopulation quantification as assessed by high-parameter flow cytometry

Subject	Arrhythmia burden (total h per d)	Graft size (% of scar size)	Graft size (% of LV)	Pre-freeze CTNT	CMs (SIRPA^+^CD90^−^)	Non-arrhythmogenic CMs (CD200^−^SIRPA^+^CD90^−^)	Arrhythmogenic CMs (CD200^+^SIRPA^+^CD90^−^)	Committed VCMs (CD77^+^CD200^−^)	Mesoderm progenitors (CD13^+^)	Fibroblasts (CD90^+^)	Endothelial cells (CD31^+^CD34^+^)	Non-myocytes (lineage unspecified)
***PSC-CM subjects***												
**PSC-CM1**	15.0	19.8	1.9	64	68.2	33.0	35.2	1.3	3.0	0.5	0.0	26.9
**PSC-CM2**	2.4	N/A	N/A	71	41.5	41.5	0.0	1.5	31.5	0.0	0.0	25.5
**PSC-CM3**	5.8	37.2	3.8	73	50.6	47.9	2.7	1.7	1.3	6.8	0.2	39.4
**PSC-CM4**	4.7	3.5	0.4	84	66.3	42.9	23.4	2.7	17.1	0.7	0.2	13.0
**PSC-CM5**	15.7	3.0	0.3	85	N/A	N/A	N/A	N/A	N/A	N/A	N/A	N/A
**Mean (±s.e.m.)**	**8.7 (2.8)**	**15.9 (8.1)**	**6.4 (0.8)**	**75.4 (4.0)**	**56.7 (6.4)**	**41.3 (3.1)**	**15.3 (8.4)**	**1.8 (0.3)**	**13.2 (7.1)**	**2.0 (1.6)**	**0.1 (0.1)**	**26.2 (5.4)**
***PSC-CM*** **+** ***AA subjects***												
**PSC-CM** **+** **AA1**	1.1	15.8	2.0	70	87.1	13.8	73.3	0.2	1.9	0.0	0.2	10.6
**PSC-CM** **+** **AA2**	0.8	42.5	3.8	85	64.6	59.7	4.9	0.9	14.6	0.5	0.1	19.3
**PSC-CM** **+** **AA3**	0.7	2.7	0.4	90	76.5	65.6	10.9	1.0	12.1	0.6	0.1	9.7
**PSC-CM** **+** **AA4**	2.2	N/A	N/A	77	32.4	28.2	4.2	0.0	46.5	1.1	0.4	19.5
**PSC-CM** **+** **AA5**	0.2	16.2	0.3	84	N/A	N/A	N/A	N/A	N/A	N/A	N/A	N/A
**Mean (±s.e.m.)**	**1.0 (0.3)**	**19.3 (8.3)**	**1.6 (0.8)**	**81.2 (4.3)**	**65.2 (11.8)**	**41.8 (12.4)**	**23.3 (16.7)**	**0.5 (0.2)**	**18.8 (9.6)**	**0.6 (0.2)**	**0.2 (0.1)**	**14.8 (2.7)**
***PSC-CM*** **+** ***CA subjects***												
**PSC-CM** **+** **CA1**	1.5	N/A	N/A	58	46.2	37.3	8.9	0.3	0.9	2.8	0.5	49.3
**PSC-CM** **+** **CA2**	0.7	N/A	N/A	90	81.1	60.9	20.3	0.0	4.9	0.5	1.0	12.5
**PSC-CM** **+** **CA3**	14.2	N/A	N/A	63	N/A	N/A	N/A	N/A	N/A	N/A	N/A	N/A
**Mean (±s.e.m.)**	**5.5 (4.4)**	**N/A**	**N/A**	**70.3 (9.9)**	**63.7 (17.4)**	**49.1 (11.8)**	**14.6 (5.7)**	**0.2 (0.2)**	**2.9 (2.0)**	**1.6 (1.1)**	**0.7 (0.3)**	**30.9 (18.4)**
***RA-PSC-CM subjects***												
**RA-PSC-CM1**	17.7^a^	8.1	0.6	72	46.9	1.8	45.1	0.0	3.0	20.9	0.4	28.7
**RA-PSC-CM2**	14.8^b^	N/A	N/A	68	44.5	0.7	43.8	0.0	3.4	1.5	0.1	50.5
**RA-PSC-CM3**	14.0^c^	10.7	2.0	60	52.0	1.1	50.9	2.0	36.8	4.4	0.2	4.7
**Mean (±s.e.m.)**	**15.5 (1.1)**	**9.4 (1.3)**	**1.3 (1.0)**	**66.7 (3.5)**	**47.8 (2.2)**	**1.2 (0.3)**	**46.6 (2.2)**	**0.7 (0.7)**	**14.4 (11.2)**	**8.9 (6.1)**	**0.2 (0.1)**	**28.0 (13.2)**

All flow data are expressed as a percentage of the total cell dose. VCM, ventricular cardiomyocytes; N/A, not available.

^a^Subject died on day 20.

^b^CA procedures were performed on day 10 and day 15.

^c^Subject died on day 10.

### Enrichment of atrial and pacemaker-like PSC-CMs

**Fig. 4:**
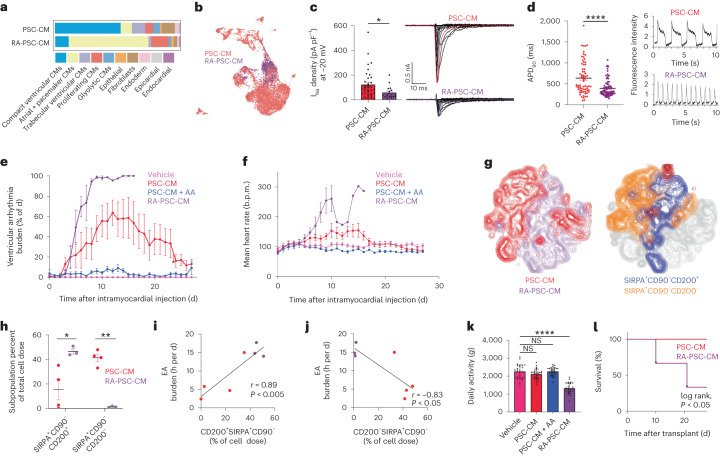
RA-PSC-CMs are enriched with atrial and pacemaker subpopulations and are highly arrhythmogenic. **a**, Contribution of cell subtypes from PSC-CMs and RA-PSC-CMs. **b**, UMAP plot of PSC-CMs (red) and RA-PSC-CMs (purple). **c**, Analysis of sodium current densities. Left, maximum current densities recorded at −20 mV in PSC-CMs (*n* = 35 biologically independent experiments) and RA-PSC-CMs (*n* = 16 biologically independent experiments) (*P = 0.02, Mann–Whitney test, two-tailed, mean ± s.e.m.). Right, sodium currents (I_Na_) in PSC-CMs and RA-PSC-CMs (inset, voltage protocol). ms, milliseconds; nA, nanoampere. **d**, Left, action potential duration at 90% depolarization (APD_90_) in PSC-CMs (*n* = 68 biologically independent experiments) and RA-PSC-CMs (*n* = 85 biologically independent experiments) (****P < 0.0001, Mann–Whitney test, two-tailed, mean ± s.e.m.). Right, action potential morphology and beat rates from PSC-CMs and RA-PSC-CMs. **e**,**f**, Percentage of time spent in ventricular arrhythmia (**e**) and mean heart rate per day between groups (**f**) (mean ± s.e.m.). (*RA-PSC-CM, *n* = 3 only from day 0 to day 10; *n* = 1 from day 11 to day 20. Pig 1 in the RA-PSC-CM group died on day 20; pig 2 in the RA-PSC-CM group underwent CA on day 10 with data excluded thereafter; pig 3 in the RA-PSC-CM group died on day 10.). **g**, High-parameter flow cytometric analysis comparing representative PSC-CM and RA-PSC-CM cell doses. Left, concatenated *t*-SNE plot showing distribution of cells from a PSC-CM dose (red) and an RA-PSC-CM dose (purple). Right, concatenated *t*-SNE plot showing distribution of SIRPA^+^CD90^−^CD200^+^ and SIRPA^+^CD90^−^CD200^−^ CMs. **h**, Proportion of SIRPA^+^CD90^−^CD200^+^ and SIRPA^+^CD90^−^CD200^−^ CMs in PSC-CM or RA-PSC-CM doses (*P = 0.02, ***P = 0.0001; unpaired *t*-test, df = 5, two-tailed; PSC-CM, *n* = 4 biologically independent animals; RA-PSC-CM, *n* = 3 biologically independent animals; mean ± s.e.m.). **i**,**j**, Correlation between arrhythmia burden and arrhythmogenic SIRPA^+^CD90^−^CD200^+^ CMs (**i**) (*r* = 0.89, *P* = 0.007, Pearson correlation) or non-arrhythmogenic SIRPA^+^CD90^−^CD200^−^ CMs (**j**) (*r* = −0.83, *P* = 0.02, Pearson correlation) in PSC-CM (red) and RA-PSC-CM (purple) cell doses. **k**, Total daily activity between groups (*g* force) (vehicle versus PSC-CM, *P* = 0.24; vehicle versus PSC-CM + AA, *P* > 0.99; vehicle versus RA-PSC-CM, ****P < 0.0001; ordinary one-way ANOVA with Dunnett’s multiple-comparison test, *n* = 28 per group, mean ± s.e.m.). **l**, Kaplan–Meier survival curve (*P = 0.04, log-rank test). Source data

**Extended Data Fig. 5:**
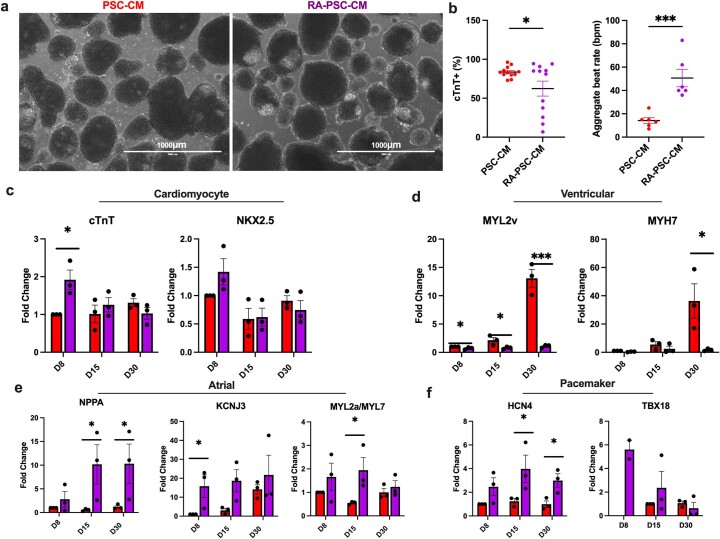
Stirred tank bioreactor generation and characterisation of RA-PSC-CMs. **(a)** Appearance of PSC-CM and RA-PSC-CMs at day 15 of bioreactor differentiation protocol. **(b)** Cardiac troponin T expression (*p = 0.04, unpaired T test, two-tailed, n = 12 biologically independent experiments, mean ± SEM). and aggregate beat rate comparison between PSC-CMs and RA-PSC-CMs (***p = 0.0009, unpaired T test, two-tailed, n = 6 biologically independent experiments, mean ± SEM). **(c)** qPCR quantified expression of general cardiomyocyte (* p = 0.02, unpaired T test, two-tailed, n = 3 biologically independent experiments, mean ± SEM), **(d)** ventricular (* p = 0.04, ***p = 0.001, unpaired T test, two-tailed, n = 3 biologically independent experiments, mean ± SEM), **(e)** atrial (*p = 0.04, unpaired T test, two-tailed, n = 3 biologically independent experiments, mean ± SEM), and **(f)** pacemaker cardiomyocyte markers (* p = 0.04, unpaired T test, two-tailed, n = 3 biologically independent experiments, mean ± SEM). Source data

To further explore the arrhythmogenic potential of atrial and pacemaker-like CMs, we developed a bioreactor differentiation protocol to enrich for these subpopulations. By modifying our standard protocol to activate the retinoic acid (RA) signaling pathway from day 2 to day 6 (Extended Data Figs. 1a and 5a), cardiac progenitors were directed to an atrial and pacemaker-like fate (RA-PSC-CM)^43^. Expression of atrial and nodal genes such as *NPPA*, *MYL7*, *SHOX2* and *HCN4* was upregulated as early as day 8 and significantly by day 15 in RA-treated cultures, with converse suppression of ventricular markers such as *IRX4* and *MYL2* (Extended Data Fig. 5d–f). This transcriptional pattern was confirmed with scRNA-seq analysis, in which RA-PSC-CMs showed a striking increase in atrial and pacemaker-like CMs and reduction of ventricular CMs (Fig. 4a,b). Electrophysiological analysis confirmed the phenotypic difference between cell preparations, with RA-PSC-CMs exhibiting faster spontaneous firing rates, reduced action potential duration and lower sodium current densities (Fig. 4c,d), features all consistent with atrial and pacemaker CMs rather than ventricular CMs.

### RA-PSC-CMs are highly arrhythmogenic

To confirm whether RA-PSC-CM transplantation would augment in vivo arrhythmia burden, transplantation studies were conducted in three further infarcted porcine subjects in the second phase of our large-animal experiments. These additional animals all received an equivalent dose of 750 million RA-PSC-CMs 2 weeks after MI. A striking increase in arrhythmia burden and rate was noted in all three animals, which experienced rapid and near-continuous EA by day 8 after cell delivery (Fig. 4e,f). High-parameter flow cytometric analysis of each cell dose showed a greater proportion of the suspected arrhythmogenic SIRPA^+^CD90^−^CD200^+^ subpopulation and a reduction in the non-arrhythmogenic SIRPA^+^CD90^−^CD200^−^ subpopulation (Fig. 4g,h). Importantly, these additional animals significantly strengthened the positive (*r* = 0.89, *P* < 0.005) and negative (*r* = −0.83, *P* < 0.05) arrhythmia correlation of these surface marker signatures (Fig. 4i,j), confirming the potential utility for these signatures in arrhythmia prediction. Elevated arrhythmia rate and burden were less favorably tolerated by the animals, which exhibited reduced activity levels as quantified by accelerometer data (Fig. 4k). Unfortunately, two of the three RA-PSC-CM recipients succumbed to arrhythmic deaths before completing their experimental time course (Fig. 4l), with the third surviving only due to timely intervention with catheter ablation (CA), described further below. Together, these data confirm the highly arrhythmogenic potential of atrial and pacemaker-like CMs and identify SIRPA^+^CD90^−^CD200^+^ and SIRPA^+^CD90^−^CD200^−^ as surface marker signatures identifying arrhythmogenic or non-arrhythmogenic cell preparations, respectively.

### RA-PSC-CM grafts comprise arrhythmogenic subpopulations

**Fig. 5:**
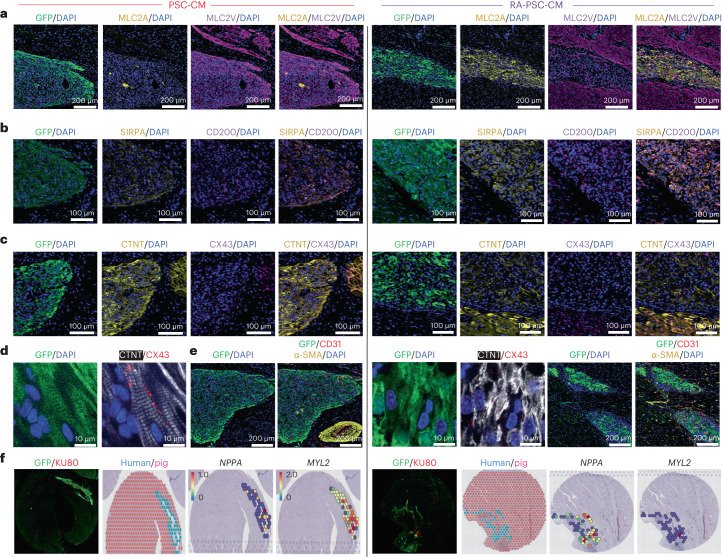
RA-PSC-CM grafts are enriched for atrial myocyte markers and show reduced sarcomeric protein and CX43 organization in comparison to PSC-CM grafts. **a**, Representative immunofluorescence images from PSC-CM- and RA-PSC-CM-treated hearts showing that PSC-CM grafts are composed almost entirely of MLC2V^+^ myocytes, in contrast to RA-PSC-CM grafts, which predominantly contain MLC2A^+^ myocytes. DAPI, 4,6-diamidino-2-phenylindole. **b**, Representative immunofluorescence images from PSC-CM- and RA-PSC-CM-treated hearts, showing increased abundance of arrhythmogenic SIRPA^+^CD200^+^ myocytes in RA-PSC-CM grafts. **c**, Low-magnification immunofluorescent images of CTNT and CX43 staining in PSC-CM and RA-PSC-CM grafts, showing increased expression of CTNT in PSC-CM grafts compared to in RA-PSC-CM grafts. In both graft types, CX43 expression is less abundant than in the surrounding pig myocardium. **d**, High-magnification confocal images of the previous grafts showing highly organized and aligned sarcomeres with appropriate localization of CX43 to the intercalated disks in the PSC-CM graft. By stark contrast, the RA-PSC-CM graft has disorganized CTNT expression, with sporadic expression of CX43. **e**, Immunofluorescent staining for CD31 and α smooth muscle actin (α-SMA) shows an abundance of neovessels in both PSC-CM and RA-PSC-CM grafts. **f**, Spatial transcriptomic analysis of PSC-CM and RA-PSC-CM grafts shows reduced expression of the gene encoding atrial natriuretic peptide (*NPPA*) and increased expression of *MYL2* or *MLC2v* in PSC-CM grafts. This phenotype is reversed in RA-PSC-CM grafts.

**Extended Data Fig. 6:**
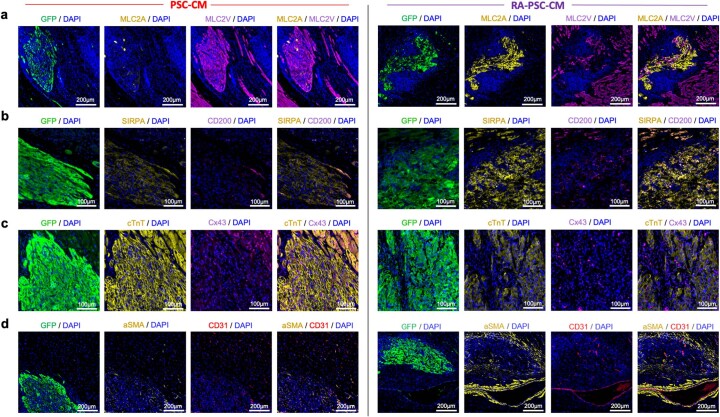
Additional PSC-CM and RA-PSC-CM graft histology. **(a)** Representative immunofluorescence images from additional PSC-CM and RA-PSC-CM treated hearts, showing PSC-CM grafts are composed almost entirely of MLC2v+ myocytes, in contrast to RA-PSC-CM grafts, which predominantly contain MLC2a+ myocytes. **(b)** Representative immunofluorescence images from PSC-CM and RA-PSC-CM treated hearts, showing increased abundance of arrhythmogenic SIRPA+/CD200+ myocytes in RA-PSC-CM grafts. **(c)** Low magnification immunofluorescent images of cardiac troponin T (cTnT) and connexin 43 (Cx43) staining in PSC-CM and RA-PSC-CM grafts, showing increased expression of cTnT in PSC-CM grafts compared to RA-PSC-CM. In both graft types, Cx43 expression is less abundant than in the surrounding pig myocardium. **(d)** Immunofluorescent staining for CD31 and alpha smooth muscle actin shows an abundance of neovessels in both PSC-CM and RA-PSC-CM grafts.

The fate of engrafted cells was probed in histological and spatial transcriptomic experiments. Human grafts were identified by staining with antibodies specific to the human nuclear antigen KU80 or by targeting the green fluorescence protein (GFP) within the gCaMP indicator of our cell line (Extended Data Fig. 6a). For detection of ventricular and atrial-like CMs within the graft, we stained sections with antibodies targeting CTNT, myosin light chain (MLC)2V and MLC2A. Interestingly, RA-PSC-CM grafts were composed of strikingly more MLC2A^+^ CMs, suggesting greater atrial CM engraftment (Fig. 5a and Extended Data Fig. 6a). In addition, these grafts also had a greater proportion of arrhythmogenic SIRPA^+^CD200^+^ CMs (Fig. 5b and Extended Data Fig. 6b).

To visualize sarcomere organization and gap junction formation between CMs, we stained grafts with antibodies specific to CTNT and connexin 43 (CX43) (Fig. 5c and Extended Data Fig. 6c). High-magnification confocal images show organized sarcomeres in standard PSC-CM grafts, with appropriate localization of CX43 to the intercalated disks (Fig. 5d). By stark contrast, RA-PSC-CM grafts had disorganized CTNT expression, with sporadic and lateralized CX43 expression patterns. We also stained tissue with antibodies for CD31 and α smooth muscle actin to identify new vessel growth within the grafts (Fig. 5e and Extended Data Fig. 6d). Both standard PSC-CM and RA-PSC-CM grafts contained abundant microvessels important in supporting long-term graft survival.

**Fig. 6:**
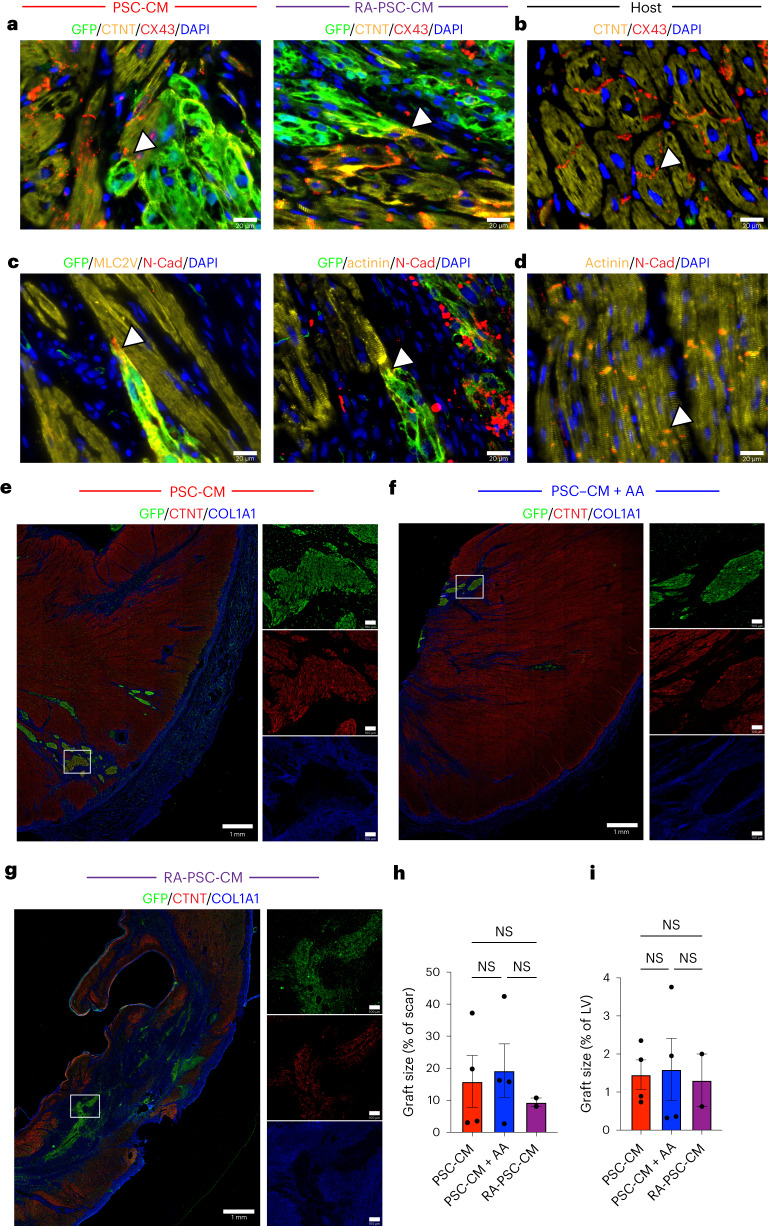
PSC-CM and RA-PSC-CM graft–host electromechanical coupling and graft size quantification. **a**, Representative immunofluorescence images from PSC-CM- and RA-PSC-CM-treated hearts, showing gap junction protein CX43 expression at the intercalated disks between GFP^+^ graft and host CMs. **b**, Positive control from host pig myocardium, showing CX43 expression at the intercalated disks. **c**, Representative immunofluorescence images from PSC-CM- and RA-PSC-CM-treated hearts, showing expression of the cell–cell adhesion protein N-cadherin (N-Cad), at the intercalated disks between GFP^+^ graft and host CMs. **d**, Positive control from host pig myocardium, showing N-cadherin expression at the intercalated disks. **e**–**g**, Representative immunofluorescence images of grafts from PSC-CM (**e**), PSC-CM + AA (**f**) and RA-PSC-CM (**g**) recipients. Insets show magnified single-channel images of GFP, CTNT or collagen type I α1 chain (COL1A1) from the labeled area of the overview image. **h**, Plot showing graft size as a percentage of infarct area in stained whole-mount sections (PSC-CM, *n* = 4; PSC-CM + AA, *n* = 4; RA-PSC-CM, *n* = 2; biologically independent animals; data are presented as mean ± s.e.m.; *P* = 0.88, Kruskal–Wallis test). **i**, Plot showing graft size as a percentage of left ventricular area in stained whole-mount sections (PSC-CM, *n* = 4; PSC-CM + AA, *n* = 4; RA-PSC-CM, *n* = 2; biologically independent animals; data are presented as mean ± s.e.m.; *P* = 0.64, Kruskal–Wallis test). Source data

We confirmed electromechanical coupling of human PSC-CM and RA-PSC-CM grafts to the porcine myocardium by identifying regions of gap junction (CX43) and adhesion protein (N-cadherin) expression at intercalated disks between graft and host CMs (Fig. 6a–d). We also stained whole-mount sections to estimate graft size as a proportion of infarct area and left ventricle (LV) area in each cell treatment group (graft area as percent of infarct: PSC-CM, 15.9% ± 8.1%; PSC-CM + AA, 19.3% ± 8.3%; RA-PSC-CM, 9.4% ± 1.3%; *P* = 0.88, NS. Graft area as percent of LV: PSC-CM, 6.4% ± 0.8%; PSC-CM + AA, 1.6% ± 0.8%; RA-PSC-CM, 1.3% ± 1.0%; *P* = 0.91, NS.) (Fig. 6e–i). There was no significant correlation between graft size and arrhythmia burden (Extended Data Fig. 4k,l).

**Extended Data Fig. 7:**
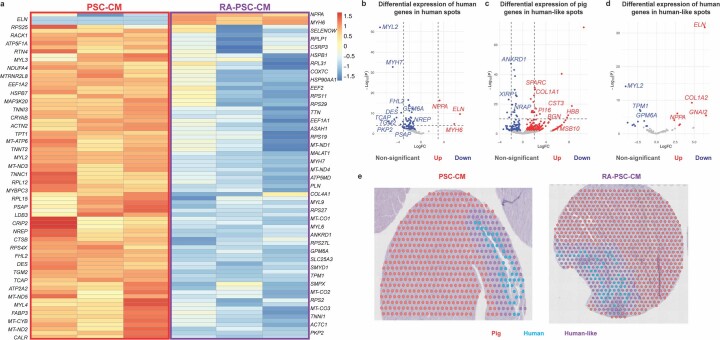
Spatial transcriptomic assessment of PSC-CM and RA-PSC-CM grafts. **(a)** Heat map of differentially expressed genes in PSC-CM (outlined by red box) and RA-PSC-CM grafts (outlined by purple box). **(b)** Comparing transcriptional signatures between spatially defined human, pig, and interfaced spots between RA treated and standard treated samples. Left - Differential expression of genes for human cells in human spots. **(c)** Differential expression of genes for pig cells in human-like interfaced spots. **(d)** Differential expression of genes for human cells in human-like interfaced spots. **(b-d)** edgeR pipeline was followed to conduct differential gene expression tests, by performing library size normalization and the quasi-likelihood test. Genes with adjusted p values less than 0.05 were considered significant **(e)** Data driven spatial mapping of human (cyan) and pig (red) tissue regions across one PSC-CM and one RA-PSC-CM recipient with unbiased identification of the interfaced spots between the grafted human cells and host pig cells. The interfaced spots (purple) are those with predominantly pig genes nearest to human spots.

**Extended Data Fig. 8:**
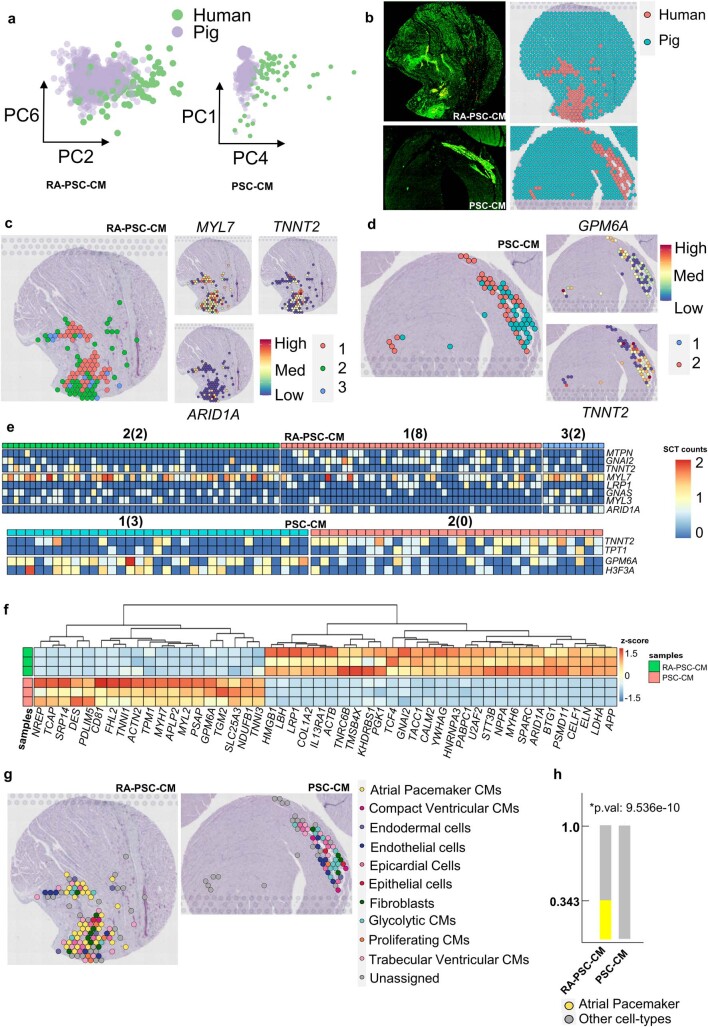
Extended spatial transcriptomic analysis of PSC-CM and RA-PSC-CM grafts, and surrounding pig myocardium. **(a)** Separation of human and pig spots by principal component analysis, using all genes as input. (**b)** Thresholding for human spots ( > 50 human genes) highly correlates with immunofluorescent staining of GFP+ human PSC-CM graft. (**c)** Unbiased clustering of human PSC-CM and RA-PSC-CM **(d)** spots and relative expression of cardiomyocyte-associated and other relevant genes **(e). (f)** Pseudobulk differential gene expression between PSC-CM and RA-PSC-CM graft shows upregulation of pan-cardiomyocyte and ventricular genes (*ACTN2*, *TNNI1*, *TNN13*, *MYH7*, *MYL2*) in PSC-CM graft and upregulation of atrial and pacemaker associated genes, calcium handling, and extracellular matrix genes (*NPPA*, *MYH6*, *CALM2*, *ELN*) in RA-PSC-CM graft. **g)** Integrated analysis/deconvolution of spatial transcriptomic and scRNAseq datasets shows location of cell subclusters within PSC-CM and RA-PSC-CM grafts. **(h)** The test of equal or given proportions confirmed the presence of engraftment arrhythmia-causing atrial and pacemaker-like subcluster myocytes were found to be located entirely within the RA-PSC-CM graft.

Finally, using an untargeted spatial transcriptomic protocol, we were able to simultaneously capture human and pig RNA from the grafted myocardium of one PSC-CM-treated pig and one RA-PSC-CM-treated pig. Both human (red dots) and pig (cyan dots) RNA was unbiasedly captured, resulting in the detection of 1,107 and 1,229 human genes and 5,501 and 9,105 pig genes (Fig. 5f and Extended Data Figs. 7 and 8). Human regions were independently confirmed by matching immunostaining targeting the human nuclear antigen KU80 (Fig. 5f).

Pseudobulk differential expression analysis of human spots showed significant upregulation of pan-CM and ventricular genes (*ACTN2*, *TNNI1*, *TNNI3*, *MYH7*, *MYL2*) in the PSC-CM graft compared to in the RA-PSC-CM graft (Extended Data Figs. 7a,b and 8f). Conversely, RA-PSC-CM grafts had increased expression of genes associated with atrial and pacemaker CMs, calcium handling and extracellular matrix (*NPPA*, *MYH6*, *CALM2*, *ELN*) (Fig. 5f and Extended Data Figs. 7b and 8f–h). Differential expression analysis of pig genes in interfaced (human-like) spots and human genes in human-like spots showed upregulation of extracellular matrix-associated genes (*COL1A1*, *SPARC*, *ELN*) around the RA-PSC-CM graft, which suggests a hypothesis that these cells interact with the surrounding myocardium in a manner distinct from that of the PSC-CM graft (Extended Data Fig. 7c–e).

To investigate cell type distribution, we performed integrated analysis of the spatial transcriptomics dataset with our scRNA-seq dataset, in which gene signatures for each of the human cell types defined from the matched scRNA-seq dataset were used to decompose the mixture of cell types in each spot. Using the robust cell type decomposition (RCTD) cellular deconvolution algorithm^44^, we mapped the predominant cell type in each human spot (Extended Data Fig. 8g). We found that the PSC-CM graft was composed of more compact and trabecular ventricular CMs, while arrhythmogenic atrial and pacemaker-like CMs were contained entirely within the RA-PSC-CM graft (Extended Data Fig. 8g,h).

### CA is feasible to treat PSC-CM EAs

**Fig. 7:**
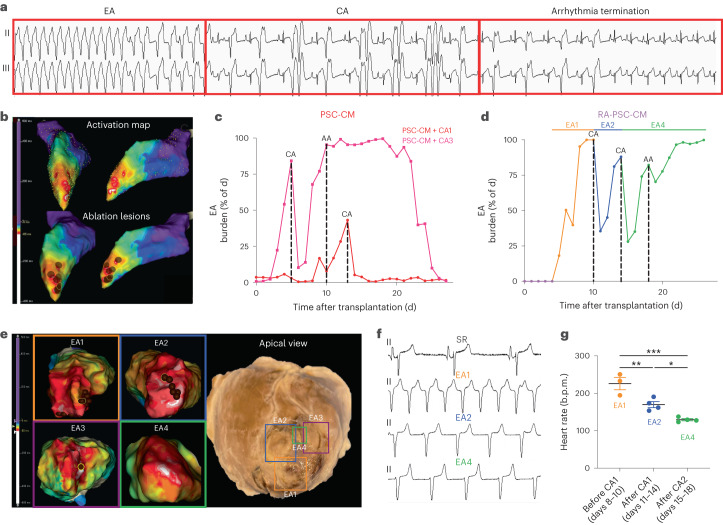
CA is a feasible and effective EA treatment strategy. **a**, Representative rhythm strip showing termination of EA during CA. **b**, Electroanatomic maps from a representative CA-treated subject. Top, activation map of EA showing anatomic origin of arrhythmia (early activation, white; late activation, purple). Bottom, activation map overlaid with ablation lesions (brown circles) that were delivered at sites of earliest activation, resulting in termination of arrhythmia. **c**,**d**, Plots depicting percentage of the day spent in ventricular arrhythmia for PSC-CM (**c**) or RA-PSC-CM (**d**) recipients treated with CA. **e**, Anatomic correlation between endocardial activation maps and extracted heart in an RA-PSC-CM subject. Left, representative activation maps in which four unique EAs were encountered. EA1–EA3 were terminated during CA1 and CA2, with final ablation lesions highlighted (brown circle with yellow outline). EA4 was persistent after CA2 and mapped at terminal EPS. Right, apical view of extracted heart following euthanasia and formalin fixation. Ablation lesions (EA1–EA3) and an island of the surviving cell graft (EA4) are outlined with excellent anatomic correlation with preceding activation maps. **f**, Representative rhythm strips from an RA-PSC-CM-treated subject of sinus rhythm (SR), EA1, EA2 and EA4. **g**, Plot depicting daily average heart rate of EA1, EA2 and EA4 in an RA-PSC-CM-treated subject (mean ± s.e.m.). Each new EA was of a significantly slower heart rate than the previously ablated EAs (*n* = 3 d for each EA; EA1 versus EA2, **P = 0.008; EA1 versus EA4, ***P = 0.0003; EA2 versus EA4, *P = 0.03; ordinary one-way ANOVA with Tukey’s multiple-comparison test). Source data

**Extended Data Fig. 9:**
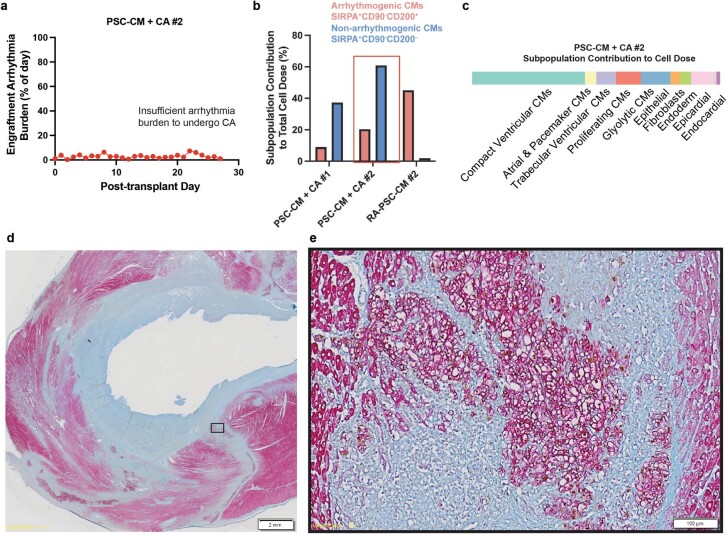
Flow cytometric and scRNA-seq profiling of PSC-CM cell dose with insufficient resultant engraftment arrhythmia burden to undergo pre-planned catheter ablation procedure. **(a)** Engraftment arrhythmia burden of PSC-CM + CA #2 following cell transplantation. **(b)** Bar plot depicting proportion of arrhythmogenic SIRPA^+^CD90^−^CD200^+^ and non-arrhythmogenic SIRPA^+^CD90^−^CD200^-^ cardiomyocytes for each subject in catheter ablation treatment group. **(c)** scRNA^−^seq analysis of cell dose received by PSC-CM + CA #2. **(d)** Whole-mount cross-section of *RA-PSC-CM* + *CA* subject. Engrafted human cardiomyocytes expressed cardiac troponin T (red) and the human-specific anti-nuclear antigen Ku80 (brown). Scarred myocardium, either from percutaneous myocardial infarction or catheter ablation, was identified with aniline blue counterstaining. **(e)** A small region of surviving human cardiomyocyte graft responsible for residual arrhythmias was identified (high magnification image of boxed region from panel (**d**)). Source data

Despite our demonstration of amiodarone–ivabradine suppressing EA burden, a fall-back strategy in the case of refractory EAs is imperative for safe PSC-CM clinical translation. Thus, in the third and final phase of our large-animal studies, we sought to determine the feasibility and efficacy of CA as an alternative EA treatment strategy. A further three pigs underwent PSC-CM delivery to enable CA testing. Of these, two subjects displayed sufficient EA to facilitate electroanatomic mapping and ablation, with inadequate burden in the third (Extended Data Fig. 9a and Supplementary Table 4). scRNA-seq for the latter showed minimal contribution of atrial and pacemaker-like CMs in the input cell dose, with flow cytometry showing a high percentage of non-arrhythmogenic SIRPA^+^CD90^−^CD200^−^ CMs (Extended Data Fig. 9b,c). Together, these findings account for the low arrhythmia burden in this pig. In the first animal, EA burden was trending upward by day 13, at which point, electroanatomic mapping and CA was performed. This localized EA origin to the inferolateral apex, and this region was targeted with a series of ablations terminating EA and restoring sinus rhythm (Fig. 7a,b). EA did not recur intraoperatively, despite a period of monitoring and aggressive arrhythmia-induction strategies (PES, isoprenaline infusion, burst pacing). Telemetry analysis from the subsequent 2 weeks showed a marked drop in EA burden (Fig. 7c), with only isolated ventricular ectopic beats rather than sustained arrhythmias noted.

In the second animal, extremely rapid EA with heart rates up to 350 b.p.m. was encountered as early as day 4 after cell injection. This was poorly tolerated, with the animal displaying clinical signs of heart failure (mottled peripheries, lethargy, poor appetite, tachypnea). As such, an emergency CA procedure was performed, and the EA was localized and successfully ablated. Interestingly, however, another slower arrhythmia immediately arose from an adjacent site. This was also successfully ablated; however, again, a new and slower arrhythmia arose from another site. Multiple unsuccessful attempts were made at ablating this third EA; however, it is likely that this final arrhythmia originated from an epicardial cell graft that could not be reached from an endocardial ablation approach. Over the subsequent days, this EA remained and eventually became continuous; however, it was of slower rate than that of the original EA and clinically was well tolerated, with the animal surviving to its final time point (Fig. 7c). Thus, CA was a life-saving intervention in this case, resolving the most aggressive EA but not resulting in complete termination of arrhythmia.

A similar finding was observed in the RA-PSC-CM recipient (Fig. 7d). Here, again, CA was a life-saving intervention facilitating survival through to the terminal time point and resolving rapid and poorly tolerated EA. However, this animal also displayed recurrent EA, and two ablation procedures were performed. Recurrent EA was always found to originate from a new site, correlating with a separate cell injection location. In total, four different origins of EAs were identified, three of which (EA1–EA3) were successfully ablated. The final arrhythmia (EA4) was mapped at the terminal procedure but was not ablated because the predetermined endpoint was reached. Examination of the heart following tissue collection demonstrated excellent anatomic correlation with preceding endocardial activation maps with clearly visible transmural ablation lesions identifying EA1–EA3 along with an island of surviving cell graft identifying EA4 (Fig. 7e). Of interest, despite recurrence following each ablation, the new arrhythmias were of lower heart rates (Fig. 7f,g) and were more favorably tolerated by the animal on clinical assessment. Histological analysis confirmed successful disruption of engrafted regions with each ablation, with intact, surviving graft responsible for the residual arrhythmia (Extended Data Fig. 9d).

Of note, oral anti-arrhythmic drugs were administered to the latter two subjects due to recurrent EA following CA (Fig. 7c,d). In both animals, however, there was no significant attenuation of arrhythmia burden despite drug therapy, highlighting the arrhythmogenicity of these particular cell doses.

In sum, these results indicate that CA is a feasible therapeutic strategy for EA; however, PSC-CM grafts may demonstrate hierarchical pacemaker-like attributes with ectopic EA activity falling back to alternative graft sites once the dominant graft has been ablated. Treatment success, particularly with highly arrhythmogenic cell doses, may require complete ablation of all engrafted regions if arrhythmogenic cell populations are not removed before transplantation.

## Discussion

The prospect of substantial cardiac remuscularization with PSC-CMs offers renewed hope to patients with heart failure^14–18^. Clinical trials are now being planned or are in progress globally. However, preclinical data informing on key safety considerations could expedite successful clinical translation. In this regard, several studies using clinically relevant large-animal models have shown that EAs are a predictable complication following intramyocardial PSC-CM delivery^8–12^. Here we show that PSC-CM-related EAs can be suppressed and potentially abolished with clinically available pharmacological and procedural therapeutics. We further identify cellular characteristics of arrhythmogenic PSC-CMs through robust phenotyping of input and engrafted cells. These data can inform cell-production strategies to provide safe and effective PSC-CMs for future clinical trials.

We show that the combination of ivabradine and amiodarone is effective in suppressing graft automaticity and reducing EA rate and burden. This result was independently reported^12^ while the current study was in progress. Ivabradine was selected, given its specific inhibition of the I_f_ current responsible for pacemaker automaticity^45–47^, a characteristic that we hypothesized may be effective in suppressing automatic EAs. Conversely, amiodarone was selected given that it is an established AA that may be empirically administered in future PSC-CM clinical trials. It has a broad mechanism of action, antagonizing K^+^, Na^+^ and Ca^2+^ channels along with β-adrenergic receptors^48^. The combination of these drugs effectively suppressed but did not eradicate EAs, necessitating assessment of alternate treatment strategies, particularly in the case of highly arrhythmogenic cell doses. Therefore, we went on to show that CA is a feasible strategy to treat EA. This proof-of-concept result has important practical implications, as the first patients are being treated with PSC-CMs. Interestingly, our ablation experiments also yielded important EA mechanistic insights, demonstrating that pacemaker activity can fall back to grafts of lower intrinsic rates once the dominant graft has been ablated. A caveat to CA therapy for EA is that ablating multiple graft regions risks loss of the contractile benefits exerted by PSC-CM transplantation. As such, we propose that CA only be pursued in the case of aggressive and pharmacologically refractory EAs and, more prudently, that the generation of less arrhythmogenic PSC-CM cell products be investigated. To this end, we also sought to gain mechanistic insights into the cellular compositions that may contribute to EAs.

Substantial variability of EA burden has been reported within and between PSC-CM large-animal studies^8–12^. Although this may be reflective of several factors such as differences in animal species, cell-delivery technique and the extent of graft integration with the host myocardium, it is likely to be predominantly determined by the molecular makeup of the cellular product^19^. Reductions in arrhythmia burden have been noted after a period of in vivo graft maturation^8,9^, suggesting that PSC-CM immaturity may be a key determinant of arrhythmogenesis^49–52^. However, not all PSC-CM recipients achieve ‘electrical maturation’ (ref. ^12^), implying that immaturity may not be the only important cellular characteristic. Current PSC-CM differentiation protocols are known to generate heterogeneous cell populations containing a mix of ventricular, atrial and pacemaker-like CMs^22,23^. We outline the importance of this heterogeneity in arrhythmogenesis, identifying atrial and pacemaker-like cells as culprit subpopulations alongside describing unique surface marker signatures predictive of arrhythmogenicity.

Cells enriched for atrial and pacemaker-like CMs were generated by addition of RA to our bioreactor differentiation protocol^53,54^ and were shown to be highly arrhythmogenic, supporting a causal link between these subpopulations and EA. We show that the enhanced automaticity of these subpopulations in vitro directly translates to more abundant and rapid EAs in vivo. Furthermore, by robustly phenotyping input cell doses and correlating these with the resultant arrhythmia burden, we identify SIRPA^+^CD90^+^CD200^+^ and SIRPA^+^CD90^−^CD200^−^ cells as populations of arrhythmogenic and potentially non-arrhythmogenic CMs, respectively. In doing so, we provide a simple quality-control tool for assessing the arrhythmogenic potential of cell doses and also a possible avenue for identifying and depleting an arrhythmogenic subpopulation before transplantation. Methods to achieve the latter will require development, optimization and iterative testing.

Interestingly, our scRNA-seq data show that these arrhythmogenic SIRPA^+^CD90^+^CD200^+^ CMs have a transcriptomic signature consistent with atrial and pacemaker subpopulations, further supporting the notion that these cell types are important in arrhythmogenesis. There is currently substantial interest in generating chamber-specific CMs for various therapeutic or drug-discovery applications^21,24,43,53,55–59^. Our data endorse the pursuit of transplanting purified ventricular CMs, devoid of atrial and pacemaker-like cells for the purposes of therapeutic cardiac remuscularization.

Of note, an important paper from the Murry group was published while our manuscript was under review^60^. Marchiano et al. were able to mitigate EAs by gene editing the sequences for four critical ion channels involved in PSC-CM automaticity. We believe that, while these authors’ approach to overcoming arrhythmogenesis differs from our own, our findings are synergistic, linking the phenotype of graft automaticity to EA burden. Of note, upon interrogation of our scRNA-seq data, we have identified that two of the three genes knocked out by these authors are differentially upregulated in the atrial and pacemaker subpopulation, *HCN4* and *SLC8A1*. This provides further validation that this culprit subpopulation is critical in arrhythmogenesis.

Finally, although not our primary intention and despite small sample sizes, we were able to demonstrate left ventricular functional improvement in cell recipients in which EAs had been ameliorated with pharmacotherapy. Although trends to improvement were noted in both the PSC-CM-alone and vehicle + AA groups, functional improvement was magnified and reached statistical significance when these therapies were combined. PSC-CM grafts elicit direct contractile force^61^, although improving PSC-CM engraftment and integration is a pending issue in the field. Our data suggest that this beneficial effect can be further enhanced once arrhythmogenicity is addressed. Further testing in appropriately powered studies will be of importance to verify this finding. Interestingly, a trend to improvement was also noted in the ungrafted right ventricle, suggesting that immunomodulatory paracrine influences may also be a factor^62^.

There are several limitations to this study. First, because the primary focus was to study EAs rather than show functional improvement, both infarct and sample sizes were small. Second, as in most other large-animal PSC-CM preclinical studies^8–11^, experiments were conducted in the sub-acute phase after MI, despite the fact that planned clinical trials are likely to be conducted in patients with chronic ischemic heart failure^18,20^. Finally, in this work, we have not transplanted cell doses devoid of the arrhythmogenic subpopulations, although this is an active pursuit of our group.

In conclusion, repopulation of the infarcted myocardium with functional, force-generating CMs is an exciting therapeutic prospect positioning PSC-CMs as a leading candidate for cardiac regeneration. Although associated with arrhythmogenesis, here, we deepen our mechanistic understanding of this predictable complication, informing the community that it is likely addressable through modifications to CM-production protocols. Additionally, we show that PSC-CM EAs can be suppressed with clinically available pharmacologic and procedural anti-arrhythmic strategies, an important safety consideration given several impending clinical trials.

## Methods

### Inclusion and ethics

All animal experiments described in this study were conducted in accordance with and approved by the Western Sydney Local Health District Animal Ethics Committee (protocol ID 4262.03.17).

The H9 cell line containing the gCaMP6f fluorescent calcium reporter was used for all experiments (provided by the University of Queensland StemCore facility). Use of this cell line and these studies were approved by the University of Queensland Human Research Ethics Committee (approval numbers HE002069 and HE000750).

### Cell production

#### Bioreactor differentiation protocol

Each production run was started from a frozen WCB cryovial, which was expanded for 8 d in monolayer on Matrigel (Corning) using the commercially available medium mTeSR Plus (Stemcell Technologies) (Extended Data Fig. 1a). The WCB was characterized for pluripotent markers, genetic stability and differentiation capacity (Supplementary Tables 3 and 5). On day −3, cells were inoculated at a density of 2.5–3.0 × 10^5^ cells per ml in mTeSR 3D medium supplemented with 10 µM Y-27632 (Tocris Bioscience) and 10 µg ml^−1^ of a thermoresponsive polymer, pNIPAM, conjugated to a fragment of recombinant fibronectin^63^ to form aggregates. A DASbox (Eppendorf) stirred tank bioreactor system was used for aggregate formation, and suspensions were controlled at 37.2 °C, pH 7.2 and 30% DO. At day −1, rapamycin (Merck) was added at a final concentration of 5 nM to enhance survival during differentiation^64^. On day 0, pluripotent aggregates were washed twice with RPMI 1640 and then cultured in RPMI B27 without insulin (Thermo Fisher Scientific) containing 6 µM CHIR 99021 (Tocris Bioscience) and 5 nM rapamycin. On day 1, 24 h after mesoderm induction, aggregates were washed once with RPMI 1640 and returned to RPMI B27 without insulin, containing 5 nM rapamycin. On day 2, aggregates were washed and returned to RPMI B27 without insulin, containing 2 µM IWP-2 (Stemcell Technologies). On day 4, aggregates were transferred to RPMI B27 with insulin, and medium was exchanged every other day until cryopreservation at day 15. Before cryopreservation, H9-gCaMP6f derived CMs were heat shocked for 30 min at 42 °C and treated with a pro-survival cocktail (100 ng ml^−1^ IGF-1, 0.6 µM cyclosporin A) to enhance their survival after transplantation^8,9,65^. CM aggregates were incubated for 6 h with 4 mg ml^−1^ collagenase IV, washed and dissociated into single cells using TrypLE (Thermo Fisher). CMs were cryopreserved at 10 × 10^6^ cells per ml in CryoStor CS10 medium (Stemcell Technologies).

#### Flow cytometry

CTNT expression was measured on a CytoFLEX Flow Cytometer (Beckman Coulter). Briefly, 1 × 10^6^ cells were fixed with 2% paraformaldehyde for 10 min at room temperature. Cells were stored in FACS wash buffer (0.5% BSA in PBS) at 4 °C until staining. Samples were permeabilized in 0.1% Triton X-100 for 10 min and then stained with anti-CTNT–FITC antibody (1:50, Miltenyi Biotec) for 30 min at room temperature. Cells were washed twice before analysis. The excitation laser and emission filters used were excitation, 488 nm; emission, 525/40 nm.

#### Quantitative PCR

After dissociation of aggregates, 1 × 10^6^ cells were collected in RNAprotect Cell Reagent (Qiagen) and stored at 4 °C until RNA extraction. RNA was extracted using the Qiagen RNeasy Mini kit according to the manufacturer’s instructions. Using the RevertAid First Strand complementary DNA (cDNA) Synthesis Kit (Thermo Fisher Scientific), 1 µg RNA was converted to cDNA in a 20-μl reaction according to the manufacturer’s instructions. cDNA was diluted 1:10 with DNase- and RNase-free water, and 1 μl of diluted cDNA used in each 10-μl reaction with 4 µl of 1 μM F and R primers and 5 μl Fast SYBR Green qPCR master mix (Thermo Fisher Scientific). Genes and the respective primers used are outlined in Supplementary Table 6. Reactions were performed in technical triplicate. qPCR was performed on the Bio-Rad CFX96 Real-Time PCR Detection System with the standard cycling parameters described in the master mix protocol. Dissociation curves were acquired at the conclusion of each run. Fold difference in expression was calculated using the comparative Ct formula, using *GAPDH* cDNA from a reference sample. Depending on the gene and time point, undifferentiated human pluripotent stem cells or fetal heart RNA were used as the reference sample.

### Single-cell RNA sequencing

#### Hashtagging and sequencing protocol

Frozen PSC-CMs were thawed in a water bath, and the contents of each vial were transferred to a 50-ml Falcon tube containing RPMI B27 medium with insulin (Thermo Fisher Scientific), 5% FBS (Thermo Fisher Scientific) and 10 µM of the ROCK inhibitor Y-27632 (Stemcell Technologies). Each sample was centrifuged at 1,000 r.p.m. for 5 min, and the cell pellet was resuspended with filtered PBS plus 2% BSA. Cells were then incubated at room temperature for 10 min in blocking buffer containing 5 µl Human TruStain FcX solution (BioLegend) plus 100 µl staining buffer (2% BSA with 0.01% Tween-20 in filtered PBS). Next, 1 µl hashtag antibody (BioLegend) was added to each sample, and samples were incubated on ice for 20 min. The hashtagging protocol employed allowed multiple samples to be pooled together in a single sequencing run. The barcode sequences for each hashtag antibody are listed in Supplementary Table 7. Cells were then washed twice with staining buffer, and 4 × 10^5^ cells from each stained sample were collected and pooled into a 2-ml Eppendorf tube. Before sequencing, a viability test suggested 80% viability of the captured cells. The cell suspensions were loaded onto 10x Genomics Single Cell 3′ Chips to form single-cell gel beads in emulsion. The Chromium library was generated and sequenced on a NextSeq 500 instrument (Illumina). Sequencing data were further processed to generate FASTQ files and the raw count matrix using the Cell Ranger pipeline at the Sequencing Facility at the University of Queensland. Multiplexed samples were demultiplexed to their original identities by mapping sample reads to the GRCh38-3.0.0 human reference genome and hashing sequences.

#### Bioinformatics analysis

The gene expression count matrix was loaded in Seurat. We removed genes that were expressed in less than 1% of total cells before creating the Seurat object. The hashtag matrix was added to the Seurat object as a new assay separate from RNA. RNA data were normalized with log normalization, and cell hashing data were normalized with centered log-ratio transformation. Following normalization, cells were demultiplexed and mapped to their original sample identities or assigned as doublets or negatives based on the Seurat HTODemux algorithm together with single-cell doublet scoring. We further filtered the cells following the standard quality-control workflow in Seurat. We removed low-quality cells after preprocessing the data and retained 11,307 cells for continued analysis. After normalization and scaling of the data, we performed dimensional reduction and selected 50 principal-component dimensions as input to the unsupervised clustering and UMAP plot. The cell type demarcating each cluster was annotated by looking into the expression level of marker genes. The Nebulosa package was used for enhanced visualization of marker gene expression.

### High-parameter flow cytometry

#### Antibody staining

Thawed PSC-CMs (~1.5 × 10^6^ cells per sample tube) were stained with an amine-reactive viability dye (Zombie NIR, BioLegend) for 30 min in PBS. The samples were washed twice with PBS + 2% FBS and centrifuged at 225*g* each time. The samples were then stained with fluorochrome-conjugated membrane marker antibodies (Supplementary Table 3) for 30 min in PBS with 2% FBS and washed twice.

For measurement of CTNT-positive cells after thawing, separate samples of PSC-CMs were fixed for 20 min with 4% PFA and permeabilized in PBS with 0.5% Tween-20. The cells were incubated with anti-human troponin T antibody in permeabilization buffer for 30 min and then washed twice with PBS + 2% FBS.

Single-color compensation controls were created using compensation beads as follows: CompBead Plus, BD, for mouse antibodies; AbC Total Antibody, Thermo Fisher, for rabbit antibodies; ArC amine-reactive beads, Thermo Fisher, for Zombie NIR and unstained PSC-CMs for endogenous gCaMP or GFP.

#### Data acquisition and analysis

PSC-CMs and compensation controls were analyzed with a BD FACSymphony A5 cytometer and FACSDiva software, using application settings.

**Extended Data Fig. 10:**
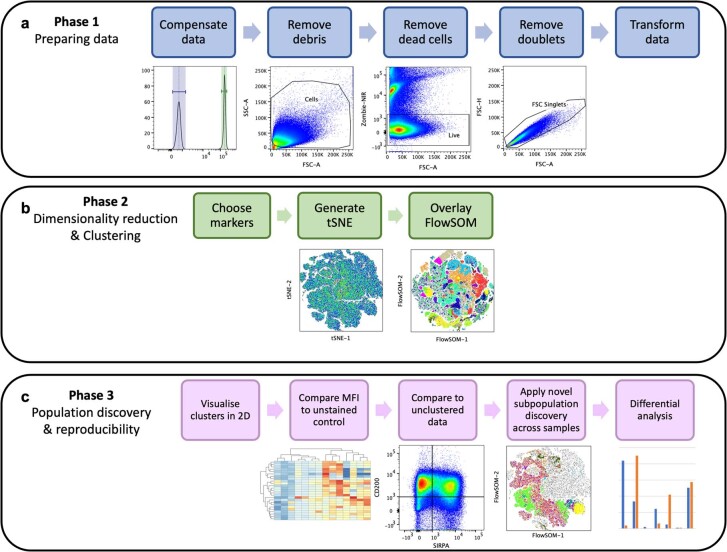
High parameter flow cytometry analysis workflow. **(a)** Steps involved in preparing data, **(b)** generating tSNE plot and overlaying with FlowSOM clusters, and **(c)** identifying and quantifying subpopulations.

Data analysis was performed using FlowJo software for Windows (BD Life Sciences, version 10.7.2). An overview of the manual gating and data-processing method is shown in Extended Data Fig. 10. Compensated data were manually gated to remove debris, non-viable cells and doublets. To identify PSC-CM subpopulations based on surface marker expression, dimensionality reduction was performed using *t*-SNE, and unsupervised clustering was performed using FlowSOM^66,67^ (opt-SNE parameters: gradient algorithm, Barnes–Hut; learning configuration, opt-SNE; KNN algorithm, exact vantage point tree; iterations, 1,000; perplexity, 30. FlowSOM parameters: number of meta-clusters, 25; SOM grid size, 10 × 10; node scale, 100%; set seed, 3.). To compare dose composition between animals and identify potentially pro-arrhythmogenic subpopulations, we downsampled and concatenated the data before dimensionality reduction and clustering.

### In vitro electrophysiology

#### iPSC-CM monolayer differentiation

iPSC-CMs (SCVI-8, Stanford Cardiovascular Institute) were used for in vitro electrophysiology experiments and were differentiated in two-dimensional monolayer culture. Two days before differentiation, PSCs were seeded into Matrigel-coated six-well culture plates at a density of 1.5 × 10^6^ cells per well in mTeSR Plus (Stemcell Technologies) with 10 µM Y-27632. On the first day of differentiation (day 0), the medium was changed from mTeSR Plus to RPMI 1640 + B27 without insulin and with GlutaMAX, penicillin–streptomycin and 6 µM of the GS3K inhibitor CHIR 99021 (Tocris Bioscience). After 24 h, the cells were washed with PBS to remove CHIR 99021, and the medium was changed to RPMI 1640 + B27 without insulin and with GlutaMAX and penicillin–streptomycin. On day 3, the medium was replenished and supplemented with the WNT signaling inhibitor IWP-2 (5 µM). On day 5, the medium was changed to RPMI 1640 + B27 without insulin and with GlutaMAX and penicillin–streptomycin. For atrial–pacemaker cell-enrichment differentiation, 1 µM RA (Sigma-Aldrich) was added at medium change on day 3 and again on day 5. On day 7, the medium was changed to RPMI 1640 + B27 with insulin, GlutaMAX and penicillin–streptomycin. Following this, the medium was changed every 2–3 d. CM beating commenced between days 6 and 7.

#### Patch clamping

For electrophysiological measurements using high-throughput patch clamping, beating iPSC-CMs were dissociated with TrypLE (Thermo Fisher) on day 15, replated onto Matrigel-coated 12-well plates at a density of 1.5 × 10^6^ cells per well and maintained in culture until use in patch-clamping experiments between days 30 and 35. On the day of patch-clamp recording, the cells were washed with PBS, dissociated with TrypLE and centrifuged for 3 min at 300*g*. The cells were then resuspended in a solution of 80% RPMI 1640 without phenol red and with Ca^2+^, 10% FBS and 20% divalent cation-free buffer containing 140 mM NaCl, 4 mM KCl, 5 mM glucose and 10 mM HEPES, pH 7.4 with NaOH. The cells were counted and diluted to a concentration of ~2 × 10^5^ cells per ml using the divalent cation-free solution.

Patch-clamp recordings were collected using a SyncroPatch 384PE (Nanion Technologies) in voltage-clamp mode. Sodium current was recorded with internal and external solutions containing the following (in mM): 110 CsF, 10 NaCl, 10 CsCl, 10 HEPES, 10 EGTA, pH 7.2 with CsOH and 140 NaCl, 4 KCl, 5 glucose, 10 HEPES, 2 CaCl_2_, 1 MgCl_2_, pH 7.4 with NaOH, respectively. Sodium current was elicited in 5-mV voltage steps ranging from −120 to +40 mV from a holding potential of –80 mV, with a 200-ms pre-pulse to −120 mV.

#### Optical electrophysiology

Individual PSC-CM action potentials were recorded using a kinetic imaging cytometer (KIC, Vala Sciences) and a voltage-sensitive fluorescent indicator (FluoVolt, Thermo Fisher). PSC-CMs were prepared for recording using a method similar to that previously described^68^. On day 14 of differentiation, beating CMs were dissociated with TrypLE (Thermo Fisher) and replated at low density (1.5 × 10^4^ cells per well) onto Matrigel-coated flat-bottom 96-well plates (Greiner CELLSTAR, Sigma-Aldrich) at a density of 1.5 × 10^4^ cells per well. The cells were maintained for a further 5–7 d. On the day of recording, cells were washed with 100 µl RPMI 1640 without phenol red and with Ca^2+^. The medium was removed and then replaced with 50 µl fresh RPMI without phenol red and with Ca^2+^, and samples were incubated at 37 °C for 60 min. The cells were then incubated for 20 min in RPMI 1640 without phenol red supplemented with FluoVolt, PowerLoad Pluronic solution and Hoechst dye. The FluoVolt solution was then removed and replaced with fresh RPMI 1640 without phenol red and with Ca^2+^. RPMI 1640 was used without supplementation to minimize background autofluorescence.

The cells were recorded on the KIC for 5 s unstimulated, followed by a 10-s stimulation at 1.0 Hz or 1.5 Hz, followed by another 5 s unstimulated. Cell segmentation and single-cell transient extraction were performed with CyteSeer Scanner software (Vala Sciences). Cell AP durations and AP upstroke velocities were obtained from KIC DAT software conversion of FluoVolt fluorescence measurements and CyteSeer cell segmentation^69^. The AP durations and activation rates of each cell are reported as the mean from all unstimulated APs recorded for that cell. APs were only included when complete measured data were available.

### Porcine experiments

#### Acclimatation and housing

All experiments were conducted in female landrace swine (2–4 months, 25–30 kg) acquired from the same local source. Animals were housed in a purpose-built large-animal research facility to which they were brought 1–2 weeks before the first scheduled procedure for acclimatization.

#### Myocardial infarction

MI was percutaneously induced by methods previously described^70^. Briefly, animals were premedicated with intramuscular ketamine (10 mg per kg), methadone (0.3 mg per kg) and midazolam (0.3 mg per kg), intubated, ventilated and maintained with inhalational isoflurane. The left coronary artery was engaged percutaneously via the right femoral artery using a 6F hockey stick guiding catheter (Medtronic). A 0.36-mm coronary guidewire (SION blue, Asahi Intecc) was delivered into the left anterior descending artery. MI was induced by occluding the mid-left anterior descending artery distal to the first diagonal branch with an inflated 2.0–3.0-mm angioplasty balloon (Boston Scientific) for 90 min. Coronary angiography performed after reperfusion confirmed vessel patency and resolution of ST elevation. Ventricular arrhythmias were treated with anti-arrhythmics and defibrillation as required.

#### Telemetry device implantation

All animals underwent telemetry transmitter implantation (easyTEL+, emka TECHNOLOGIES) under general anesthesia. The transmitter was implanted in a subcutaneous pocket that was created in the left flank. Subcutaneous leads were tunneled to capture the cardiac apex and base. Signal quality was assessed before securing the device and leads in situ.

#### Telemetry analysis

Telemetric ECG and accelerometer data were continuously monitored from the time of device implantation. Semi-automated quantification of heart rate, arrhythmia burden and accelerometer data was performed offline by an expert cardiologist using the device-specific software package (ecgAUTO 3.5.5.16, emka TECHNOLOGIES). Arrhythmia was defined as an ectopic beat (for example, premature ventricular contraction) or rhythm. The entire recorded dataset for each subject was analyzed, with data presented as daily averages (mean ± s.e.m.). For each subject, a library of QRS morphologies was created and determined as either sinus rhythm or arrhythmia by the cardiologist based on features such as atrioventricular dissociation, QRS width or fusion beat. This methodology is consistent with existing literature in the field^12,60^.

#### Cardiac magnetic resonance imaging

Animals were premedicated with intramuscular ketamine (10 mg per kg), methadone (0.3 mg per kg) and midazolam (0.3 mg per kg) before intubation and mechanical ventilation. General anesthesia was induced with intravenous propofol (2–5 mg per kg) and maintained with 2% inhaled isoflurane. Breathing was held in end expiration for all image acquisitions. All CMR examinations were performed on a Siemens 3T Prisma system (Siemens Medical Systems) using an 18-channel body array together with spine array coils and four-lead ECG gating. Axial and coronal true fast imaging with steady-state precession (TrueFISP) sequences through the heart were acquired to plan preliminary two-chamber, short-axis and four-chamber single-slice gradient images. True two-chamber, three-chamber, four-chamber and right ventricular outflow tract 8-mm single-slice true fast imaging cines were then planned and acquired as well as a short-axis stack of 14 contiguous 8-mm slices, starting just beyond the apex and extending into the atrium, planned from two- and four-chamber cine images in end diastole.

TrueFISP imaging was used for all acquisitions with the following parameters: TE (echo time), 1.3 ms; TR (repetition time) at the R–R interval of the individual animal; field of view, 370 mm; slice thickness, 8 mm; in-plane resolution, 1.4 mm × 1.4 mm; flip angle, 10°; 25 calculated phases.

LGE images were acquired for tissue characterization using a segmented inversion recovery fast gradient echo sequence in SAX, LVLA and four-chamber planes. Time of inversion scout was performed at 9 min after contrast to select the appropriate inversion time (usually around 300 ms) for LGE acquisition, which began at around 11 min after injection. The SAX was run with 30 contiguous 4-mm-thick slices, covering the entire heart with an in-plane resolution of 1.5 mm × 1.5 mm, whereas, for the LVLA and four-chamber sequences, single 8-mm-thick slices were acquired with an in-plane resolution of 1.4 mm × 1.4 mm. Other LGE parameters were TR, R–R interval of the individual animal; TE, 1.65 ms; flip angle, 20°.

Image analysis was performed offline using dedicated software (Medis Suite MR 3.2, Medis Medical Imaging) by two independent, blinded cardiologists. Volumetric assessment was performed according to the Society for Cardiovascular Magnetic Resonance guidelines^71^. In brief, short-axis end-diastolic and end-systolic images were chosen as the maximal and minimal mid-ventricular cross-sectional areas. End-diastolic epicardial and endocardial borders, along with end-systolic endocardial borders, were manually traced for each slice. The vendor-specific automated contouring algorithm was not used due to suboptimal performance in non-human subjects. Papillary muscles were included in volume calculations and excluded in mass calculations. The difference between end-diastolic and end-systolic endocardial borders represented the left or right ventricular stroke volume, and ejection fraction was calculated as stroke volume/end-diastolic volume.

Infarct size was quantified using a separate dedicated software (Segment, Medviso). Short-stack late gadolinium-enhanced images were used for infarct quantification. Endocardial and epicardial borders were manually contoured from LV base to apex. The Segment automated algorithm for scar quantification was then applied by designating the left anterior wall as the infarct territory. This algorithm delineates areas of late gadolinium enhancement (LGE) as well as areas of microvascular obstruction. Infarct size was expressed in milliliters. Infarct percent was then calculated by dividing infarct size by end-diastolic left ventricular mass, as measured in end diastole on the cine short-stack acquisitions.

#### ADAS 3D CMR reconstructions

Following CMR image acquisition, a separate offline segmentation software, ADAS 3D (Galgo Medical), was used to process three-dimensional reconstructions of the LV and fibrosis as identified by LGE for integration into the electroanatomic mapping system (EAM). Complete cardiac anatomy including the coronary arteries was also exported to assist with registration to EAM reconstructions. Endocardial and epicardial borders were delineated in all slices, and an assessment of the scar was made based on the pixel signal intensity (PSI) between the two borders. Values for the identification of dense fibrosis and border zone regions using the maximum PSI have been previously correlated with low-voltage regions and conducting channels on the EAM^72,73^. Core scar was defined as 60% of maximal PSI, and border zone was defined as 40%. Fibrosis area was calculated by averaging the PSI of the endocardial to mid-myocardial layers and the average PSI of the mid-myocardial to epicardial layers^74^. The calculated values were then projected, using the trilinear interpolation method, to the LV reconstruction^74^ of the endocardial and epicardial surfaces.

#### Thoracotomy, epicardial mapping and cell injection

On day 0, animals were sedated and returned to the operating theater for thoracotomy and transepicardial injections by methods we have previously described^75^. Following cannulation and intubation, an arterial line was inserted into the auricular or distal limb artery to enable continuous blood pressure monitoring. A 100-µg fentanyl patch was applied, and intravenous antibiotic (cefazolin, 1 g) was administered. Intercostal nerve block of the 2nd to 6th rib spaces was achieved with bupivacaine and lignocaine.

Thoracotomy was performed by making an incision in the left lateral chest wall at the 4th and 5th intercostal space. The incision was opened under direct vision using self-retaining rib retractors. The pericardium was opened anterolaterally, and the apex and anterior LV were gently exposed by packing beneath the LV with wet gauze. Hemodynamics were carefully monitored, and metaraminol boluses were administered as required to maintain systolic blood pressure greater than 100 mmHg. The epicardial surface of the LV was electroanatomically mapped using an electrophysiological mapping catheter (Navistar ThermoCool SmartTouch, Biosense Webster). Cardiac MRI was imported to the EAM system, and registration was performed by placing the mapping catheter at fiducial landmarks under direct visualization. Fiducial landmarks used included the apex, the mitral valve annulus and the left anterior descending artery. EAM points were acquired on the EAM system with the mapping catheter, and identical landmarks were selected on the cardiac MRI. An initial registration was performed with landmarks, and then a secondary registration with a best fit of all anatomy was applied using EAM system software (CartoMerge, Biosense Webster)^76^. Confirmation of registration accuracy was reconfirmed with the EAM catheter. Following registration, the electroanatomic voltage map was assessed to identify scar, border and remote zones. Transepicardial injections of either vehicle (8 × 300-µl injections of RPMI B27) or cells (750 × 10^6^ cells distributed in 8 × 300-µl injections) were then performed under direct vision into portions of infarct and border zones using a 27-gauge insulin syringe. All pigs were treated with transcutaneous fentanyl patches (100 µg per h) and intravenous buprenorphine (150–300 µg every 8–12 h) for up to 72 h post-operatively for analgesia.

#### Central venous catheter insertion and immunosuppression

All subjects received a three-drug immunosuppressive regimen to prevent xenograft rejection. Commencing 5 d before cell injections, animals received oral cyclosporine A (10−15 mg per kg twice daily), aiming to maintain trough levels greater than 250 ng ml^−1^. On day 0, a 2-lumen 5-French (Fr) central venous catheter (Teleflex) was implanted in the right jugular vein under ultrasound guidance to facilitate ongoing administration of intravenous immunosuppressants and regular blood draws. Following central line placement and before cell injection, 500 mg abatacept (CTLA4-Ig, Bristol Myers Squibb), 30 mg per kg methylprednisone and 3–5 mg per kg cyclosporine A were administered intravenously. From day 1 after transplantation until euthanasia, subjects received oral cyclosporine A (10–15 mg per kg) twice daily to maintain trough levels greater than 250 ng ml^−1^, along with 100 mg intravenous methylprednisone daily. Another 250-mg dose of intravenous abatacept was given 2 weeks after transplantation. Prophylactic oral amoxicillin–clavulanic acid was given daily to all subjects to prevent central line and thoracotomy infections, and 30 mg oral lansoprazole was given daily for gastrointestinal protection.

#### Anti-arrhythmic treatment

Animals randomized to anti-arrhythmic treatment received a 150-mg i.v. amiodarone bolus at the time of cell injection. From day 1 after transplantation until euthanasia, they were treated with a regimen of 200 mg oral amiodarone twice daily and 10 mg oral ivabradine twice daily.

#### Electrophysiological study

Before euthanasia, all subjects underwent electrophysiological examination under general anesthesia to determine inducibility, mechanism and electroanatomic origin of any ventricular arrhythmia. An 8.5-Fr Agilis steerable introducer (Abbott Medical) was inserted into the right common femoral vein under ultrasound guidance, through which an Advisor HD Grid (Abbott Medical) multi-electrode electrophysiological catheter was advanced to the right ventricle. A 6-Fr decapolar electrophysiological catheter was positioned into the coronary sinus via the right femoral vein. The EnSite Precision EAM system (Abbott Medical) was used to generate a substrate map delineating scar, border and remote zones using bipolar and unipolar voltage cutoffs of 0.5–1.5 mV and 3–8.3 mV, respectively^77,78^. The LV was then mapped via a retrograde aortic approach. A substrate map was initially created if the subject was in sinus rhythm. Following substrate mapping, VT induction via PES was performed. A drive train of eight beats at 400 ms was followed by up to four extrastimuli delivered one at a time. Initial extrastimuli were delivered at a coupling interval of 300 ms, which was then decreased by 10 ms until ventricular refractoriness. A modified ‘Michigan’ protocol was additionally performed^79^. The Michigan protocol uses exclusively four extrastimuli. At each drive train cycle length of 350 ms, programmed stimulation is initiated with coupling intervals of 290, 280, 270 and 260 ms for the first through fourth extrastimuli. The coupling intervals of the extrastimuli are shortened simultaneously in 10-ms steps until an extrastimuli is refractory or an arrhythmia is induced. Both PES protocols were repeated at two sites. Following PES, an isoprenaline bolus (20 µg) and infusion (6–10 µg min^−1^) were administered with burst pacing starting from 300 ms and reduced in 20-ms steps until refractory during isoprenaline delivery and washout^80^. The mechanism of any spontaneous or induced ventricular arrhythmias was elucidated through standard pacing maneuvers followed by EAM to identify arrhythmia origin.

#### Catheter ablation

Subjects who underwent CA for treatment of EA were premedicated with intramuscular ketamine (10 mg per kg), methadone (0.3 mg per kg) and midazolam (0.3 mg per kg), intubated, ventilated and maintained with inhalational isoflurane. An arterial line was inserted into the auricular or distal limb artery to enable continuous blood pressure monitoring. All subjects were in spontaneous EA at the time of the ablation procedures. Arrhythmia origin was electroanatomically mapped as described above. At the site of earliest activation mapping, radiofrequency ablation lesions were delivered using a 4-mm-tip open-irrigation catheter (FlexAbility, Abbott Medical). A single grounding patch was placed on each animal, and ablation was performed in power-control mode using 30–40 W of power and an irrigation flow rate of 13 ml min^−1^ with normal saline. Delivery of each lesion was attempted for 30–60 s unless terminated prematurely due to catheter movement or impedance rise. Ablations were terminated after sinus rhythm was restored, followed by an attempt at VT induction as described above.

#### Euthanasia and tissue collection

Following the terminal EPS procedure, subjects were euthanized with potassium chloride (75–150 mg per kg) under deep anesthesia, and hearts were excised and fixed in 10% neutral buffered formalin for subsequent analysis.

### Histology

#### Tissue processing

Pig hearts were fixed whole in 10% neutral buffered formalin for a minimum of 48 h. The ventricles were then sliced into transverse sections approximately 1 cm thick from the apex (level 1) toward the base (level 7) and fixed in fresh formalin for a further 48 h. After fixation, the tissues were transferred to 70% ethanol, processed and paraffin embedded. Processing, embedding and sectioning of level 1 blocks were performed at the Westmead Institute for Medical Research. Processing, embedding and sectioning of large blocks (level 2 and above) were performed by Veterinary Pathology Diagnostics Services at the University of Sydney.

#### Immunohistochemistry

Immunohistochemistry analyses were performed on 4-µm sections taken from level 1, 2 or 3. For graft size measurements, we stained LV whole-mount sections from level 2 or 3 with primary antibodies targeting human KU80, GFP, COL1A1 and CTNT. Sections were deparaffinized in xylene and hydrated by sequential incubation in 100%, 95%, 70% and 50% ethanol and water. Antigen retrieval was performed using heated sodium citrate buffer, and the sections were then washed with PBS + 0.1% Tween-20, blocked with 5% goat serum in PBS with 0.05% Tween-20 and stained with primary antibodies overnight at 4 °C (Supplementary Table 8). The following day, the sections were washed and incubated with secondary antibodies (Supplementary Table 8) for 1 h at room temperature in the dark. The sections were then washed again and incubated with DAPI (1 µg ml^−1^, Sigma-Aldrich–Merck) for 10 min, rinsed with PBS and mounted with PBS–glycerol.

For demonstration of PSC-CM grafts and radiofrequency ablation sites, whole-mount sections from level 2 tissue blocks were stained with primary antibodies targeting human KU80 and CTNT as described above. For brightfield detection of secondary staining, we used the ImmPRESS Duet Double Staining HRP/AP Polymer Kit (Vector Laboratories, MP-7714) and then counterstained sections with aniline blue (1% aniline blue in Milli-Q water for 1 min).

#### Imaging and analysis

Immunofluorescence and brightfield microscopy were performed on an Olympus VS120 Slide Scanner, with the ×20 objective (UPLSAPO 20X/NA 0.75, WD 0.6/CG Thickness 0.17) or the ×40 objective (UPLSAPO 40X/NA 0.95, WD 0.18/CG Thickness 0.11–0.23). Images were acquired using Olympus VS-ASW 2.92 software and processed using Olympus VS-DESKTOP 2.9.

Confocal images of pig tissues immunostained for GFP, CX43 and CTNT were taken on a Leica SP8 microscope using tunable white light and 405-nm lasers. Filters were adjusted for optimal signal detection of Alexa Fluor 488, Alexa Fluor 594 and Alexa Fluor 647 using a two-sequence scanning approach. *Z* stacks (7 µm with a step size of 0.5 mm) were imaged using a confocal pinhole set at 1 airy unit.

#### Graft size measurement

Images for whole-mount sections acquired with the Olympus VS120 were analyzed for graft quantification with ImageJ. The scar area was obtained by thresholding the COL1A1 channel and removing pericardium collagen. The ‘Analyse Particles’ function was used to exclude interstitial collagen. Whole-tissue area was obtained by thresholding the CTNT channel and adding the CTNT channel to the COL1A1 channel using ‘Image calculator’ to represent the full tissue. ‘Analyse Particles’ was used to exclude background noise. The polygon selection tool was used to manually select graft area for quantification. Graft size was presented as a percentage of whole-LV area or a percentage of scar area.

### Spatial transcriptomics

#### Tissue preparation

Formalin-fixed paraffin-embedded tissue from level 1 blocks was used for spatial transcriptomics analysis of engrafted PSC-CMs. Using a tissue microarray punch, we collected samples of tissue 3 mm in diameter from regions confirmed to contain PSC-CM grafts. The samples were melted down and re-embedded into four 6-mm × 6-mm paraffin blocks, each containing three samples.

#### RNA quality assessment

Three to five 7-μm sections were collected per sample for RNA extraction using the RNeasy FFPE Kit (73504, Qiagen). RNA integrity number and DV_200_ were determined by Bioanalyzer using the RNA 6000 Pico Kit (5067-1513, Agilent). DV_200_ is the percentage of total RNA fragments greater than 200 bp in length. All samples had DV_200_ from 46% to 57%.

#### Tissue optimization

To find the optimal permeabilization time and tissue-sectioning thickness, we adapted the Visium Spatial Tissue Optimization User Guide for fresh-frozen ST (CG000238 Rev A, 10x Genomics). FFPE blocks were sectioned at 7 μm with a rotary microtome. Paraffin sections were floated on nuclease-free water in a 43 °C water bath for 2 min per section and captured while floating to each allocated array on Visium Tissue Optimization slides (3000394). The slides with FFPE sections were then dehydrated with silica bead desiccants at room temperature for 1 h before overnight storage at 4 °C in a sealed slide box containing silica beads. The following day, slides were dried at 37 °C for 15 min for complete dehydration before wax melting at 60 °C for 60 min and deparaffinization with xylene (5 min, twice). Tissue rehydration was performed using an ethanol gradient (100% for 2 min, twice; 90% for 2 min, twice; 85% for 2 min). The above temperatures and times were optimized to ensure that tissue adhered to the slide throughout the process.

Tissue sections were stained with hematoxylin and eosin following the standard protocol and imaged at ×20–40 magnification on a Zeiss AxioScan Z1 slide scanner. Next, tissues were decross-linked by incubation with collagenase for 20 min at 37 °C and then at 70 °C in TE buffer (pH 8.0) for 60 min. Immediately after decross-linking, tissue sections were permeabilized by incubation with pepsin (0.1%) for different amounts of time (5–40 min). In this step, RNA was released from the tissue onto the glass slide, where poly(A) tails hybridize to the slide-bound poly(dT) oligonucleotides. After permeabilization, a reverse-transcription reaction was performed to synthesize cDNA labeled with cyanine 3. Tissue sections were then removed from the slide using tissue-removal buffer. The cDNA products on the slide were visualized with a Leica DMi8 inverted widefield microscope. By comparing fluorescence intensity and assessing signal diffusion, we determined the optimal permeabilization conditions to avoid over- or under-permeabilization.

#### Sequencing library preparation

FFPE blocks were sectioned and placed onto a Visium Spatial Gene Expression Slide (2000233, containing four capture areas), dried, dewaxed, deparaffinized and stained as in the tissue optimization procedure. After permeabilization, cDNA was synthesized using standard unlabeled nucleotides, followed by second-strand synthesis. Spatial barcodes and unique molecular identifiers (UMIs) are part of the oligonucleotides printed on the glass slide, and this sequence is incorporated into the first-strand cDNA. The double-stranded DNA products were denatured, and the released cDNA was amplified by PCR for 18 cycles, end repaired, A tailed and size selected with SPRIselect (0.8× bead cleanup). Adaptor ligation, PCR amplification and sample indexing were performed following the Illumina TruSeq protocol. The final PCR amplification (10−15 cycles) and size selections (0.55× and 0.8× double-sided cleanup) were performed before quality control by the Bioanalyzer. Libraries were sequenced with the NovaSeq SP100 version 1.5 kit (138 cycles) and a paired-end protocol as follows: read 1, 28 bp; index 1, 10 bp; index 2, 10 bp; read 2, 90 bp.

#### Sequencing data analysis

The raw NovaSeq BCL output file was converted to a FASTQ file using Space Ranger mkfastq version 1.3.0 and bcl2fastq2 version 2.17.1. The FASTQ files were processed further to remove adaptor sequences and poly(A) sequences in read 2 using cutadapt version 3.2. A hybrid reference genome was made by combining Sscrofa11.1 (reference annotation Sscrofa11.1.105.gtf) with GRCh38-3.0.0 (genome build GRCh38.p12) using Space Ranger mkref. The trimmed FASTQ reads were mapped to the hybrid genome using Space Ranger count, which is based on STAR splicing-aware alignment. The gene expression matrix contains uniquely and confidently mapped UMIs. A UMI was only counted if it was mapped to a single exonic locus (at least 50% of the read intersects an exon) or, if multiple mapping occurred, this UMI needed to have MAPQ255 (uniquely mapped) and bases 100% compatible with the exon of an annotated transcript and aligned to the same strand. High-resolution hematoxylin and eosin images were used for mapping gene expression to spatial spots. Gene expression was used for downstream analysis.

#### Quality control

It was an expected challenge to simultaneously capture interspecies gene expression from a fixed engrafted tissue in a way that both mRNA from human and pig could be measured. We developed a protocol that allows unbiased capture of the poly(A) mRNA from these two species. We noted that the quality of the overall data was suboptimal. Therefore, we applied an unbiased quality-control process to select the top two samples with the highest quality among all samples. Before downstream analysis, genes and spots that were likely noise or outliers were identified and removed based on the distribution of gene and spot statistics. Across all samples, we established the same threshold on the minimum number of human genes detected per spot (80) and the minimum number of spots that a gene was detected (ten). This process resulted in two samples that passed our quality threshold, one with RA treatment (RA-PSC-CM3) and the other with standard treatment (PSC-CM + CA2). Each sample contained data for 1,229 and 1,107 human genes and 9,105 and 5,501 pig genes, from 808 and 567 spots for RA and standard treatments, respectively.

#### Identification of human and pig spots

For each spot, we calculated its human score as the total number of reads that were mapped to the human genome and its pig score as that mapped to the pig genome. In tissue with RA-PSC-CM treatment, we assigned a spot as a human one if its human score was >30 and its pig score was <700. In tissue with standard treatment, we assigned a spot as a human one if its human score was >30 and its pig score was <350. The thresholds were defined based on density distributions of values across all spots. Principal-component analysis of all spots based on all human and pig genes clearly distinguished human and pig spots (Extended Data Fig. 8a). In total, we identified 62 human spots in the RA-PSC-CM sample and 50 human spots in the PSC-CM sample. Spot assignment was consistent with experimental immunofluorescent evidence (Extended Data Fig. 8b). Based on physical spatial distance, we further classified a pig spot as a human-like interfaced spot if this pig spot were in the 20 nearest neighbor of a human spot and its distance to the human spot was less than 1,500 pixels (Extended Data Fig. 7e). These interfaced (human-like) spots could dominantly contain pig cells and a minority of human cells and clearly represented the neighborhood where cellular interactions could happen through mechanisms such as paracrine ligand–receptor signaling.

#### Differential expression analysis

After quality control, although there was only one biological sample for each condition, we used the observation that spots that were measured separately from each other and independent spots can represent different histology in one tissue section (for example, different cell type composition). We therefore performed pseudobulk differential gene expression analyses. For each sample, we randomly pooled spots into three equally sized pools. For each pool, an average gene expression was calculated spot-wise. By doing this, we created three pseudo-replicates for each condition, RA treatment versus standard treatment. With the pseudo-replicates, we performed differential expression analysis following the edgeR pipeline, using library size normalization and the quasi-likelihood test. Genes with adjusted *P* value (FDR adjusted) less than 0.05 were considered significant.

#### Deconvolution and cell type analysis

As each spatial spot in the spatial transcriptomics can contain approximately one to nine cells, we performed decomposition analysis to decompose the cell type mixture in each spot. We used our matched scRNA-seq dataset, as the cell types identified in the scRNA-seq data from the same project are highly suitable to be used as the reference to infer cell type composition in the matched spatial transcriptomics data. We applied the top-performing RCTD deconvolution algorithm^44^ and identified 11 detailed categories of cell type representation (Extended Data Fig. 8g). To independently assess the results from the deconvolution, we performed clustering analysis using all genes to group similar spots into two to three general categories that were distinct by transcriptional signatures (Extended Data Fig. 8c–f). The clustering approach is, therefore, independent of a reference scRNA-seq dataset. We then assessed the consistency between the general categories found by clustering with the detailed cell types inferred by deconvolution. The spatial distributions are highly consistent between the two methods (Extended Data Fig. 8c,d).

### Statistical analyses

Continuous data are presented as mean ± s.e.m. Normality was assessed using the Shapiro–Wilk test, with appropriate parametric or nonparametric tests performed depending on the distribution of data. Statistical comparisons of normally distributed data were conducted using unpaired *t*-tests or ordinary one-way ANOVA followed by Sidak’s post hoc test to adjust for multiple comparisons. Non-normal data were statistically compared using the Mann–Whitney or the Kruskal–Wallis test followed by Dunn’s test to adjust for multiple comparisons. Correlations were expressed using Pearson’s or Spearman’s correlation coefficients. Survival analyses were performed using the Kaplan–Meier method, and the log-rank test was applied to determine significance between overall survival between groups. *P* values < 0.05 were considered statistically significant. All analyses were performed using GraphPad Prism version 9.3.1 software.

Representative images displayed in figures were selected from a pool of reproducible experiments conducted on a minimum of three biologically independent samples.

### Reporting summary

Further information on research design is available in the Nature Portfolio Reporting Summary linked to this article.

### Supplementary information

Reporting Summary

Supplementary Tables Supplementary Table 1: Ventricular arrhythmia characteristics. Supplementary Table 2: CMR scar size and volumes for phase 1 large-animal experiments. Supplementary Table 3: Antibodies used for high-parameter flow cytometry experiments. Supplementary Table 4: Radiofrequency ablation parameters for subjects with CA. Supplementary Table 5: Characteristics of the working cell bank. Supplementary Table 6: Genes and primers used for qPCR experiments. Supplementary Table 7: scRNA-seq hashtag barcode sequences. Supplementary Table 8: Antibodies used for immunohistochemistry experiments.

### Source data

Source Data Figs. 1, 2, 4, 6 and 7 and Extended Data Figs. 1, 4, 5 and 9 Numerical source data for Figs. 1, 2, 4, 6 and 7 and Extended Data Figs. 1, 4, 5 and 9.

## Data Availability

All data supporting the findings in this study are included in the main article and its associated files. All scRNA-seq and spatial transcriptomics data can be found under accession number GSE248953. Source data are provided with this paper.

## Code availability

Code for the KIC DAT software can be made available upon request to A. Hill, Victor Chang Cardiac Research Institute (a.hill@victorchang.edu.au).
