# Supplementary material for: Cellular heterogeneity of pluripotent stem cell-derived cardiomyocyte grafts is mechanistically linked to treatable arrhythmias
Source: Nat Cardiovasc Res. 2024 Feb 6;3(2):145–65. doi: 10.1038/s44161-023-00419-3 (PMC11358004; doi:10.1038/s44161-023-00419-3)
Supplement: Supplementary file 1 — Reporting Summary [file 44161_2023_419_MOESM1_ESM.pdf]

nature portfolio

Corresponding author(s): James J.H. Chong

Last updated by author(s): Dec 5, 2023

Reporting Summary

Nature Portfolio wishes to improve the reproducibility of the work that we publish. This form provides structure for consistency and transparency in reporting. For further information on Nature Portfolio policies, see our Editorial Policies and the Editorial Policy Checklist.

Statistics

For all statistical analyses, confirm that the following items are present in the figure legend, table legend, main text, or Methods section.

n/a	Confirmed	
☐	☒	The exact sample size (*n*) for each experimental group/condition, given as a discrete number and unit of measurement
☐	☒	A statement on whether measurements were taken from distinct samples or whether the same sample was measured repeatedly
☐	☒	The statistical test(s) used AND whether they are one- or two-sided<br>*Only common tests should be described solely by name; describe more complex techniques in the Methods section.*
☐	☒	A description of all covariates tested
☐	☒	A description of any assumptions or corrections, such as tests of normality and adjustment for multiple comparisons
☐	☒	A full description of the statistical parameters including central tendency (e.g. means) or other basic estimates (e.g. regression coefficient) AND variation (e.g. standard deviation) or associated estimates of uncertainty (e.g. confidence intervals)
☐	☒	For null hypothesis testing, the test statistic (e.g. *F*, *t*, *r*) with confidence intervals, effect sizes, degrees of freedom and *P* value noted<br>*Give P values as exact values whenever suitable.*
☒	☐	For Bayesian analysis, information on the choice of priors and Markov chain Monte Carlo settings
☒	☐	For hierarchical and complex designs, identification of the appropriate level for tests and full reporting of outcomes
☐	☒	Estimates of effect sizes (e.g. Cohen's *d*, Pearson's *r*), indicating how they were calculated

Our web collection on statistics for biologists contains articles on many of the points above.

Software and code

Policy information about availability of computer code

Data collection

All software used for data collection has been described in the methods section of the manuscript. Specifically:
Large animal telemetry data was acquired using Emka IOX2 v2.10.8.6 software (Emka technologies, Paris, France).
Cardiac Magnetic Resonance Imaging data was acquired using a Siemens Prism 3T system running on Siemens Medical Systems software {Siemens Healthineers, Pennsylvania, U.S.A).
Large animal electroanatomical mapping data was acquired using CARTO3 v6 mapping software (Biosense Webster, Calfornia, U.S.A).
Flow cytometry data was acquired using BD FACSDiva Software v8.5 (BD Biosciences, New Jersey, U.S.A).

Data analysis

All software used for data analysis has been described in the methods section of the manuscript. Specifically:
Large animal telemetry data was analysed using Emka ecgAUTO analysis software v3.5.5.16 (Emka technologies, Paris, France).
Cardiac Magnetic Resonance Imaging data was analysed using using Medis Suite MR v3.2 (Medis Medical Imaging, Schuttersveld 9, The Netherlands), and Segment v4.0 R11044c (Medviso, Lund, Sweden) & ADAS-3D (Galgos Medical, Barcelona, Spain) analysis softwares.
Large animal electroanatomical mapping data was analysed using CARTO3 v6 mapping software (Biosense Webster, Calfornia, U.S.A).
Single cell RNA sequencing analysis was performed using Seurat v4 {Satjialab).
Spatial transcriptomics analysis was performed using Space Ranger mkfastq v1.3.0 and Cell Ranger v7.0 (10x Genomics,
Flow cytometry data was analysed using FlowJo v10.7.2 (BD, Oregon, U.S.A).
Optical electrophysiology data was analysed with CyteSeer v2.0 software (Vala sciences, California, U.S.A).
Optical action potential measurements were analysed using custom KIC data analysis software (KICDAT, VCCRI, NSW, Australia).
Statistical analysis was performed using GraphPad v9.3.1 software (Dotmatics, California, U.S.A).

For manuscripts utilizing custom algorithms or software that are central to the research but not yet described in published literature, software must be made available to editors and reviewers. We strongly encourage code deposition in a community repository (e.g. GitHub). See the Nature Portfolio guidelines for submitting code & software for further information.

Data

Policy information about availability of data

All manuscripts must include a data availability statement. This statement should provide the following information, where applicable:

- Accession codes, unique identifiers, or web links for publicly available datasets
- A description of any restrictions on data availability
- For clinical datasets or third party data, please ensure that the statement adheres to our policy

All data supporting the findings in this study are included in the main article and associated files. Source data are provided with this manuscript. All scRNA-seq and spatial transcriptomics data can be found at https://www.ncbi.nlm.nih.gov/geo/query/acc.cgi?acc=GSE248953 (accession number: GSE248953).

Research involving human participants, their data, or biological material

Policy information about studies with human participants or human data. See also policy information about sex, gender (identity/presentation), and sexual orientation and race, ethnicity and racism.

Reporting on sex and gender	n/a
Reporting on race, ethnicity, or other socially relevant groupings	n/a
Population characteristics	n/a
Recruitment	n/a
Ethics oversight	n/a

Note that full information on the approval of the study protocol must also be provided in the manuscript.

Field-specific reporting

Please select the one below that is the best fit for your research. If you are not sure, read the appropriate sections before making your selection.

☒ Life sciences ☐ Behavioural & social sciences ☐ Ecological, evolutionary & environmental sciences

For a reference copy of the document with all sections, see nature.com/documents/nr-reporting-summary-flat.pdf

Life sciences study design

All studies must disclose on these points even when the disclosure is negative.

Sample size	At the time this study was being designed, the experiments described were being done for the first time. No pre-specified effect size could be determined a priori. However, for our phase 1 studies, a sample size of 4 subjects per treatment group has 80% power (at 0.05 alpha) to detect an 80% reduction in arrhythmia burden.
Data exclusions	As described in the manuscript, 3 animals were excluded from cardiac magnetic resonance imaging (CMR) functional analysis due to small or absent myocardial infarct creation, a pre-specified exclusion criteria for this endpoint. This data is reported in Supplementary Table 3.
Replication	The nature of large animal work does not readily lend itself to performing multiple batches of experiments. However, as described, our results were quite consistent within treatment groups. As described in the manuscript, analysis of CMR data from phase 1 studies was extremely reproducible, with excellent inter- and intra-observer variability. All CMR data was analysed by two blinded observers with excellent inter-observer variability. One observer completed analysis of all CMR studies on two occasions, at least two weeks apart with excellent intra-observer variability.
Randomization	Animals in phase 1 experiments were randomly allocated to treatment groups by a computer generated randomization algorithm. Animals in phase 2 and 3 studies were not randomized as these were proof-of-concept experiments to test the arrhythmogenicity of RA-PSC-CM cell doses and efficacy of catheter ablation to treat engraftment arrhythmias. However, all experimental methodology and variables such as animal age, sex, weight, infarct creation and immunosupression protocol were controlled and consistent within each group.
Blinding	All research personnel involved in the large animal experiments were blinded to treatment allocations during experimentation. In addition, all investigators involved in telemetry and CMR data analysis were blinded to treatment allocation at the time of data analysis.

Reporting for specific materials, systems and methods

We require information from authors about some types of materials, experimental systems and methods used in many studies. Here, indicate whether each material, system or method listed is relevant to your study. If you are not sure if a list item applies to your research, read the appropriate section before selecting a response.

Materials & experimental systems

n/a	Involved in the study
☐	☒ Antibodies
☐	☒ Eukaryotic cell lines
☒	☐ Palaeontology and archaeology
☐	☒ Animals and other organisms
☒	☐ Clinical data
☒	☐ Dual use research of concern
☒	☐ Plants

Methods

n/a	Involved in the study
☒	☐ ChIP-seq
☐	☒ Flow cytometry
☒	☐ MRI-based neuroimaging

Antibodies

Antibodies used	All flow cytometry, scRNA-seqe and immunohistochemistry antibodies and dilutions used are listed in Supplementary Tables 3,7 and 8.
Validation	Each antibody was validated using established positive and negative controls and titered for an optimal signal:noise ratio. Both human and porcine samples were used to establish which antibodies to use and the specifics of their immunostaining assays.

Eukaryotic cell lines

Policy information about cell lines and Sex and Gender in Research

Cell line source(s)	H9 gCaMP6f human ESC line -StemCore, University of Queensland<br>SCVI 8 human iPSC line - Stanford Cardiovascular Institute Biobank
Authentication	H9 gCaMP6f line -See Supplementary Table 1. Pluripotency via flow cytometry (>90% positive for Oct4, Sox2, Tra-1-60, SSEA4) and immunofluorescence (>80% positive for Oct4, Nanog, and Tra-1-60). Karyotyped with Stem Cell Technologies Human PSC Genetic Analysis Kit (#07550) -negative for 8 common deletions and duplications found in human PSCs. Differentiation capacity via flow cytometry for cTnT expression (>80% positive for cTnT at day 15 of differentiation)
Mycoplasma contamination	H9 gCaMP6f line -Lonza Mycoalert mycoplasma detection assay-negative result.<br>SCVI 8 line - Lonza Mycoalert mycoplasma detection assay - negative result.
Commonly misidentified lines<br>(See ICLAC register)	No commonly misidentified cell lines were used in this study.

Animals and other research organisms

Policy information about studies involving animals; ARRIVE guidelines recommended for reporting animal research, and Sex and Gender in Research

Laboratory animals	All experiments were conducted in female landrace swine (2 to 4 months, 25 -30kg) acquired from the same local source.
Wild animals	No wild animals were used in this study.
Reporting on sex	All experiments were conducted in female swine.
Field-collected samples	No field collected samples were used in this study.
Ethics oversight	All procedures in this study were approved by the Western Sydney Local Health District Animal Ethics Committee (protocol ID: 4262.03.17).

Note that full information on the approval of the study protocol must also be provided in the manuscript.

Plants

Seed stocks: n/a

Novel plant genotypes: n/a

Authentication: n/a

Flow Cytometry

Plots

Confirm that:

☒ The axis labels state the marker and fluorochrome used (e.g. CD4-FITC).

☒ The axis scales are clearly visible. Include numbers along axes only for bottom left plot of group (a 'group' is an analysis of identical markers).

☒ All plots are contour plots with outliers or pseudocolor plots.

☒ A numerical value for number of cells or percentage (with statistics) is provided.

Methodology

Sample preparation: Please see methods for detailed information.
The expression of cTnT was measured on a Cytoflex Flow Cytometer (Beckman Coulter). Briefly, 1 x 106 cells were fixed with 2% paraformaldehyde for 10 min at room temperature. Cells were stored in FACS wash buffer (0.5% BSA in PBS) at 4 •c until staining. Samples were permeabilised in 0.1% Triton X-100 for 10 min then stained with CTNT-FITC 1:50 (Miltenyi Biotec) for 30 min at room temperature. Cells were washed twice before analysis. The excitation laser and emission filters used were: Ex. 488, Em. 525/40.
High parameter flow cytometry:
Thawed PSC-CMs (~1.Sx106 cells per sample tube) were stained with an amine reactive viability dye (Zombie NIR, Biolegend) for 30 minutes in PBS. The samples were washed twice in PBS+ 2% FBS and centrifuged at 225G each time. The samples were then stained with fluorochrome-conjugated membrane marker antibodies (Supplementary Table 4) for 30 minutes in PBS + 2% FBS and washed twice.
Single colour compensation controls were created using compensation beads as follows: CompBead Plus, BO, for mouse antibodies; AbC Total Antibody, ThermoFisher, for rabbit antibodies; ArC amine reactive beads, ThermoFisher, for Zombie NIR and unstained PSC-CMs for endogenous gCaMP/GFP.

Instrument: Beckman Coulter Cytoflex Flow Cytometer
BD FACSymphony AS

Software: Acquisition - BD FACSDiva v8.5
Analysis -FlowJo vl0.7.2

Cell population abundance: Refer to Supplementary Table 5. Raw data files with cell numbers in each sub-population and median fluorescence intensities for all markers also available on request.

Gating strategy: Compensated data was manually gated to remove debris, non-viable cells, and doublets. To identify PSC-CM sub-populations based on surface marker expression, dimensionality reduction was performed using t-distributed stochastic neighbour embedding (tSNE), and unsupervised clustering was performed using FlowSOM (opt-SNE parameters: Gradient algorithm - Barnes-Hut, Learning configuration -opt-SNE, KNN algorithm -exact vantage point tree, Iterations -1000, Perplexity-30; FlowSOM parameters: Number of meta-clusters -25, SOM grid size -lOxlO, Node scale -100%, Set Seed -3). To compare dose composition between animals and identify potentially pro-arrhythmogenic sub-populations, we downsampled doses to 200,000 events and concatenated the data prior to dimensionality reduction and clustering.
Refer to Extended Data Figure 10 for an overview of this process.

☒ Tick this box to confirm that a figure exemplifying the gating strategy is provided in the Supplementary Information.
