# Supplementary material for: Cellular heterogeneity of pluripotent stem cell-derived cardiomyocyte grafts is mechanistically linked to treatable arrhythmias
Source: Nat Cardiovasc Res. 2024 Feb 6;3(2):145–65. doi: 10.1038/s44161-023-00419-3 (PMC11358004; doi:10.1038/s44161-023-00419-3)
Supplement: Supplementary file 2 — Supplementary Table 1: Ventricular arrhythmia characteristics. Supplementary Table 2: CMR scar size and volumes for phase 1 large-animal experiments. Supplementary Table 3: Antibodies used for high-parameter flow cytometry experiments. Supplementary Table 4: Radiofrequency ablation parameters for subjects with CA. Supplementary Table 5: Characteristics of the working cell bank. Supplementary Table 6: Genes and primers used for qPCR experiments. Supplementary Table 7: scRNA-seq hashtag barcode sequences. Supplementary Table 8: Antibodies used for immunohistochemistry experiments. [file 44161_2023_419_MOESM2_ESM.pdf]

**Supplementary Table 1. Ventricular arrhythmia characteristics**

| Subject | Re-entrant arrhythmia inducible at terminal EPS | Automatic arrhythmia present at terminal EPS | CMR assessed scar size (%) | Longest run of arrhythmia identified on telemetry analysis (hours) |
|---|---|---|---|---|
| | | *Sham* | | |
| *Sham #1* | No | No | 0 | n/a |
| *Sham #2* | No | No | 1.2 | n/a |
| | | *Vehicle* | | |
| Vehicle #1 | Yes | No | 10.9 | n/a |
| Vehicle #2 | Yes | No | 7 | n/a |
| Vehicle #3 | Yes | No | 14.7 | n/a |
| Vehicle #4 | No | No | 1.3 | n/a |
| Vehicle #5 | No | No | 0 | n/a |
| | | *Vehicle + AA* | | |
| Vehicle + AA #1 | Yes | No | 16.0 | n/a |
| Vehicle + AA #2 | No | No | 13.6 | n/a |
| Vehicle + AA #3 | Yes | No | 11.8 | n/a |
| Vehicle + AA #4 | No | No | 0.8 | n/a |
| | | *PSC-CM* | | |

| | | | | |
|---|---|---|---|---|
| PSC-CM #1 | Yes | Yes | 7.1 | 24 |
| PSC-CM #2 | Yes | Yes | 11.8 | 7 |
| PSC-CM #3 | No | Yes | 0.2 | 17 |
| PSC-CM #4 | Yes | No | 15.4 | 12 |
| PSC-CM #5 | Yes | Yes | 15.2 | 22 |
| | | *PSC-CM + AA* | | |
| PSC-CM + AA #1 | Yes | No | 19.8 | 3 |
| PSC-CM + AA #2 | No | No | 9.8 | 4 |
| PSC-CM + AA #3 | Yes | No | 25.2 | 1 |
| PSC-CM + AA #4 | Yes | No | 22.8 | 1 |
| PSC-CM + AA #5 | No | No | 6.0 | 1 |
| | | *RA-PSC-CM* | | |
| RA-PSC-CM #1 | n/a | n/a | n/a | 72 |
| RA-PSC-CM #2 | n/a | n/a | n/a | 24 |
| RA-PSC-CM #3 | Yes | No | n/a | 66 |

**Supplementary Table 2. CMR scar size and volumes for Phase 1 large animal experiments.**

| Subject | Timepoint | Scar Size (% of LV mass) | LVEDV (mL) | LVESV (mL) | LVSV (mL) | LVEF (%) | RVEF (%) |
|---|---|---|---|---|---|---|---|
| | | | *Sham* | | | | |
| **Sham #1** | Baseline | 0 | 77 | 31 | 46 | 59 | 56 |
| | Follow-up | 0 | 86 | 35 | 51 | 59 | 56 |
| **Sham #2** | Baseline | 1.2 | 77 | 34 | 43 | 57 | 58 |
| | Follow-up | 1.6 | 85 | 37 | 48 | 57 | 58 |
| **Mean (± SEM)** | **Baseline** | **0.6 (0.6)** | **77 (0)** | **32.5 (1.5)** | **44.5 (1.5)** | **58 (1.0)** | **57 (1.0)** |
| | **Follow-up** | **0.8 (0.8)** | **85.5 (0.5)** | **36 (1.0)** | **49.5 (1.5)** | **58 (1.0)** | **57 (1.0)** |
| | | | *Vehicle* | | | | |
| **Vehicle #1** | Baseline | 8.9 | 95 | 53 | 42 | 44 | 45 |
| | Follow-up | 10.9 | 109 | 61 | 48 | 44 | 44 |
| **Vehicle #2** | Baseline | 6.6 | 71 | 39 | 32 | 46 | 61 |
| | Follow-up | 7.0 | 100 | 55 | 45 | 46 | 52 |
| **Vehicle #3** | Baseline | 14.7 | 102 | 63 | 39 | 39 | 52 |
| | Follow-up | 13.1 | 123 | 74 | 49 | 40 | 56 |
| **Mean (± SEM)** | **Baseline** | **10.1 (2.4)** | **89.3 (9.4)** | **51.7 (7.0)** | **37.7 (3.0)** | **43 (2.1)** | **52.7 (4.6)** |

| | | | | | | | |
|---|---|---|---|---|---|---|---|
| | **Follow-up** | **10.3 (1.8)** | **110.7 (6.7)** | **63.3 (5.6)** | **47.3 (1.2)** | **43.3 (1.8)** | **50.7 (3.5)** |
| | | | *Vehicle + AA* | | | | |
| **Vehicle + AA #2** | Baseline | **16.0** | **82** | **49** | **33** | **40** | **61** |
| | Follow-up | **12.7** | **109** | **57** | **52** | **47** | **71** |
| **Vehicle + AA #3** | Baseline | **13.6** | **134** | **90** | **44** | **33** | **67** |
| | Follow-up | **20.0** | **143** | **90** | **53** | **37** | **61** |
| **Vehicle + AA #4** | Baseline | **11.8** | **94** | **43** | **51** | **55** | **65** |
| | Follow-up | **6.7** | **131** | **74** | **57** | **59** | **68** |
| **Mean (± SEM)** | **Baseline** | **13.8 (1.2)** | **103.3 (15.7)** | **60.7 (14.8)** | **42.7 (5.2)** | **42.7 (6.5)** | **64.3 (1.8)** |
| | **Follow-up** | **13.1 (3.8)** | **127.7 (10.0)** | **73.7 (9.5)** | **54 (1.5)** | **47.7 (6.4)** | **66.7 (3.0)** |
| | | | *PSC-CM* | | | | |
| **PSC-CM #1** | Baseline | 12.1 | 89 | 59 | 30 | 33 | 54 |
| | Follow-up | 7.1 | 105 | 60 | 45 | 42 | 59 |
| **PSC-CM #2** | Baseline | 10.6 | 105 | 60 | 45 | 43 | 48 |
| | Follow-up | 11.8 | 126 | 70 | 56 | 44 | 61 |
| **PSC-CM #4** | Baseline | 13.4 | 109 | 68 | 41 | 38 | 60 |
| | Follow-up | 15.4 | 134 | 80 | 54 | 41 | 73 |

| | | | | | | | |
|---|---|---|---|---|---|---|---|
| **PSC-CM #5** | Baseline | 15.2 | 99 | 58 | 41 | 42 | 55 |
| | Follow-up | 13.9 | 138 | 67 | 71 | 51 | 56 |
| **Mean (± SEM)** | **Baseline** | **12.8 (1.0)** | **100.5 (4.3)** | **61.3 (2.8)** | **39.3 (4.5)** | **39.0 (2.3)** | **54.3 (2.4)** |
| | **Follow-up** | **11.8 (1.8)** | **125.8 (7.4)** | **69.2 (5.8)** | **56.3 (3.4)** | **44.5 (2.3)** | **62.3 (3.7)** |
| | | | *PSC-CM + AA* | | | | |
| **PSC-CM + AA #1** | Baseline | 22.2 | 108 | 76 | 32 | 31 | 51 |
| | Follow-up | 19.8 | 133 | 87 | 46 | 35 | 60 |
| **PSC-CM + AA #2** | Baseline | 9.8 | 106 | 50 | 56 | 53 | 59 |
| | Follow-up | 9.8 | 113 | 37 | 76 | 67 | 64 |
| **PSC-CM + AA #3** | Baseline | 20.3 | 149 | 108 | 41 | 28 | 45 |
| | Follow-up | 25.2 | 215 | 142 | 73 | 34 | 53 |
| **PSC-CM + AA #4** | Baseline | 22.8 | 121 | 74 | 47 | 39 | 58 |
| | Follow-up | n/a | 162 | 86 | 76 | 47 | 54 |
| **PSC-CM +AA #5** | Baseline | 6.0 | 85 | 49 | 36 | 42 | 58 |
| | Follow-up | 1.6 | 118 | 45 | 73 | 62 | 64 |
| **Mean (± SEM)** | **Baseline** | **16.2 (3.5)** | **113.8 (10.5)** | **71.4 (10.8)** | **42.4 (4.2)** | **38.6 (4.4)** | **54.2 (2.7)** |
| | **Follow-up** | **14.1 (5.2)** | **148.2 (18.8)** | **79.4 (18.7)** | **68.8 (5.7)** | **49.0 (6.8)** | **59.0 (2.4)** |

| | | | | | | | |
|---|---|---|---|---|---|---|---|
| | | | *Excluded Subjects (Scar size < 1.5%)* | | | | |
| **Vehicle #4** | Baseline | 1.3 | 108 | 60 | 48 | 44 | 56 |
| **Vehicle #5** | Baseline | 0 | 77 | 36 | 41 | 53 | 64 |
| **Vehicle + AA #1** | Baseline | 0.8 | 66 | 28 | 38 | 58 | 64 |
| **PSC-CM #3** | Baseline | 0.2 | 83 | 45 | 39 | 47 | 57 |
| **Mean (± SEM)** | **Baseline** | **0.6 (0.3)** | **83.5 (8.9)** | **42.2 (6.9)** | **41.5 (2.3)** | **50.5 (3.1)** | **60.3 (2.2)** |

**Supplementary Table 3. Antibodies used for high-parameter flow cytometry experiments.**

| Company | Marker | Clone | Fluorophore | Dilution | Significance |
|---|---|---|---|---|---|
| | GCaMP | | eGFP | | Genetically encoded, intracellular calcium transient marker[1] |
| **BD Biosciences** | VCAM-1 | 51-10C9 | PerCP-Cy5.5 | 1:100 | hPSC-CM lineage marker[2] SIRPA$^{+}$/VCAM1$^{+}$ phenotype indicates committed CM[3] |
| **Biolegend** | SIRPα | SE5A5 | PECy5 | 1:100 | hPSC-CM marker[3, 4] |
| **ThermoFisher Scientific** | Desmoglein 2 | CSTEM28 | PE | 1:200 | Desmosomal protein, highly expressed in CMs[5] |
| **Biolegend** | CD31 | WM59 | PECy7 | 1:100 | Endothelial cell marker[6] |
| **BD Biosciences** | CD235a | GA-R2(HIR2) | BUV395 | 1:100 | Early marker of mesoderm that gives rise to ventricular CM's[7] |
| **Biolegend** | CD90 | 5E10 | BV650 | 1:100 | Cardiac fibroblast marker, although non-specific (also expressed on endothelium, mesenchymal stem cells)[8] |

| | | | | | |
|---|---|---|---|---|---|
| **Biolegend** | CD77 | 5B5 | BV510 | 1:100 | $CD77^+/CD200^-$ suggests ventricular CM phenotype[9, 10] |
| **Biolegend** | CD13 | WM15 | BV786 | 1:100 | Early cardiac mesoderm marker[11] |
| **Biolegend** | CD200 | OX-104 | BV421 | 1:100 | $CD77^+/CD200^-$ suggests ventricular CM phenotype[9, 10] |
| **Biolegend** | CD34 | 581 | AF700 | 1:100 | Endothelial cell and progenitor marker[3, 6] |
| **Abcam** | Vinculin | EPR8185 | AF647 | 1:200 | Adhesion marker, links CM contractile apparatus to adherens junctions (intracellular)[12] |
| **BD Biosciences** | Cardiac Troponin T | 13-11 | BV421 | 1:20 | A regulatory protein of the cardiomyocyte contractile apparatus and pan-CM marker (intracellular)[13] |
| **Biolegend** | Zombie Viability | | NIR | 1:1000 | Live/dead stain |

**Supplementary Table 4. Radiofrequency ablation parameters for catheter ablation subjects.**

| | | | | | |
|---|---|---|---|---|---|
| | | *PSC-CM + CA #1 (Day 13 post-injection)* | | | |
| **Ablation number** | **Impedence – start (Ω)** | **Impedence – end (Ω)** | **Power (W)** | **Duration (sec)** | **Notes** |
| **1** | 170 | 162 | 30 | 40 | |
| **2** | 180 | 165 | 30 | 30 | |
| **3** | 175 | 160 | 30 | 60 | VF induced – 3x 250J shocks required, successfully defibrillated to EA with 3rd shock |
| **4** | 165 | 200 | 30 | 24 | |
| **5** | 170 | 205 | 30 | 18 | |
| **6** | 170 | 204 | 30 | 24 | |
| **7** | 170 | 158 | 30 | 30 | |
| **8** | 168 | 150 | 30 | 21 | Sinus rhythm restored |
| | | ***PSC-CM + CA#3 (Day 5 post-injection)*** | | | |
| **1** | n/a | 231 | 40 | 21 | |
| **2** | n/a | 153 | 40 | 18 | VF induced – successfully defibrillated with 200J shock |

| | | | | | |
|---|---|---|---|---|---|
| **3** | 205 | 199 | 40 | 28 | VF induced – successfully defibrillated with 200J shock |
| **4** | 175 | 202 | 40 | 22 | VF induced – successfully defibrillated with 200J shock |
| **5** | 179 | 178 | 40 | 15 | |
| **6** | 170 | 158 | 40 | 19 | VF induced – successfully defibrillated with 200J shock |
| **7** | 180 | 158 | 40 | 15 | VF induced – successfully defibrillated with 200J shock |
| **8** | 170 | 156 | 40 | 9 | VF induced – successfully defibrillated with 200J shock |
| **9** | 190 | 171 | 40 | 8 | |
| **10** | 170 | 160 | 40 | 13 | |
| **11** | 180 | 167 | 40 | 20 | VF induced – successfully defibrillated with 200J shock |
| **12** | 175 | 165 | 40 | 16 | |
| **13** | 160 | 168 | 40 | 19 | |

| | | | | | |
|---|---|---|---|---|---|
| **14** | 195 | 147 | 40 | 18 | |
| **15** | 155 | 196 | 40 | 18 | VF induced – successfully defibrillated with 200J shock |
| **16** | 155 | 169 | 40 | 16 | VF induced – successfully defibrillated with 200J shock |
| **17** | 165 | 165 | 40 | 14 | |
| **18** | 166 | 152 | 30 | 23 | |
| **19** | 155 | 163 | 30 | 15 | VF induced – successfully defibrillated with 200J shock |
| **20** | 160 | 143 | 30 | 20 | VF induced – successfully defibrillated with 200J shock |
| **21** | 156 | 143 | 30 | 20 | |
| **22** | 155 | 145 | 30 | 26 | |
| **23** | 168 | 152 | 30 | 12 | VF induced – successfully defibrillated with 200J shock |
| **24** | 166 | 143 | 30 | 22 | VF induced – successfully defibrillated with 200J shock |

| | | | | | |
|---|---|---|---|---|---|
| **25** | 155 | 156 | 30 | 26 | VF induced – successfully defibrillated with 200J shock |
| *RA-PSC-CM #3 – Catheter Ablation 1 for EA 1 (Day 10 post-injection)* | | | | | |
| **1** | 220 | 237 | 40 | 15 | Impedance cut-off |
| **2** | 190 | 165 | 40 | 30 | |
| **3** | 200 | 165 | 40 | 30 | |
| **4** | 220 | 157 | 40 | 30 | VF induced – successfully defibrillated with 250J shock back to EA1 |
| **5** | 158 | 242 | 40 | 25 | |
| **6** | 161 | 259 | 40 | 14 | |
| **7** | 170 | 246 | 40 | 12 | |
| **8** | 160 | 249 | 40 | 27 | |
| **9** | 206 | 197 | 40 | 30 | |
| **10** | 167 | 240 | 40 | 17 | |
| **11** | 164 | 247 | 40 | 17 | |
| **12** | 173 | 156 | 40 | 30 | |
| **13** | 153 | 158 | 40 | 14 | |

| | | | | | |
|---|---|---|---|---|---|
| **14** | 162 | 156 | 40 | 30 | |
| **15** | 160 | 149 | 40 | 30 | |
| **16** | 180 | 154 | 40 | 30 | Sinus rhythm restored |
| *RA-PSC-CM #3 – Catheter Ablation 2 for EA 2 and EA 3 (Day 14 post-injection)* | | | | | |
| **1** | 209 | 182 | 40 | 20 | |
| **2** | 182 | 171 | 40 | 40 | VF induced – successfully defibrillated with 250J shock back to EA2 |
| **3** | 237 | 188 | 40 | 40 | Sinus rhythm restored however onset of new EA (EA3) shortly after |
| **4** | 246 | 159 | 40 | 16 | VF induced – successfully defibrillated with 250J shock back to EA3 |
| **5** | 167 | 153 | 40 | 40 | Sinus rhythm restored |
| **6** | 160 | 185 | 40 | 19 | Empiric ablation |
| **7** | 160 | 152 | 40 | 38 | Empiric ablation |

**Supplementary Table 5. Characterisation of working cell bank**

| Characterisation | Method | Result |
|---|---|---|
| **Pluripotency** | Flow cytometry | >90% positive for each of the markers Oct4, Sox2, Tra-1-60 and SSEA4 |
| | Immunofluorescence | >80% positive for Oct4, Nanog and Tra-1-60 |
| **Mycoplasma** | Lonza Mycoalert Mycoplasma detection assay | Negative |
| **Karyotype** | Stem Cell Technologies Human Pluripotent Stem Cells Genetic Analysis Kit (Product No. #07550) | Negative for 8 common deletions and duplications found in human pluripotent cells |
| **Quantitative evaluation of differentiation capacity to cardiomyocytes** | Suspension based differentiation using small molecules | Positive detection (>80%) ^ of CM specific cardiac troponin using flow cytometry and ICC. |
| **Post thaw viability** | N/A | Confluent T75 5 days after thaw of cryopreserved vial. |

^ one-off measurement of cardiac troponin to establish suitability of working cell bank for cardiomyocyte differentiation.

**Supplementary Table 6. Genes and primers used for qPCR experiments.**

| Gene | Forward Primer | Reverse Primer |
|---|---|---|
| **OCT3/4** | AGCGATCAAGCAGCGACTAT | AGAGTGGTGACGGAGACAGG |
| **NANOG** | ACCTTCCAATGTGGAGCAAC | GAGAATTTGGCTGGAACTGC |
| **DNMT3B** | TCCTCAAAGAGTTGGGCATAA | TTTGATATTCCCCTCGTGCT |
| **HAND1** | GCCTAGCCACCAGCTACATC | ATCCGCCTTCTTGAGTTCAG |
| **GATA6** | AGAGCACCAATCCCGAGAAC | GCACGGAGGACGTGACTT |
| **EOMES** | ACCCCCTTCCATCAAATCTC | CCATGCCTTTTGAGGTGTCT |
| **MESP1** | AGCCCAAGTGACAAGGGACAACT | AAGGAACCACTTCGAAGGTGCTGA |
| **Brachyury (T)** | CCTTGCTCACACCTGCAGTAGC | GGCCAACTGCATCATCTCCA |
| **ISL1** | GAAGGTGGAGCTGCATTGGTTTGA | TAAACCAGCTACAGGACAGGCCAA |
| **SIRPA** | ACCTGGCTCAGGCTAGTTCCAAAT | TGTGCACACGTATGTGCTGTCTCT |
| **CTNT** | TTCACCAAAGATCTGCTCCTCGCT | TTATTACTGGTGTGGAGTGGGTGTGG |
| **MYH6** | TCAGCTGGAGGCCAAAGTAAAGGA | TTCTTGAGCTCTGAGCACTCGTCT |
| **MYL7** | ACATCATCACCCATGGAGACGAGA | GCAACAGAGTTTATTGAGGTGCCC |

| | | |
|---|---|---|
| **MYH7** | TCGTGCCTGATGACAAACAGGAGT | ATACTCGGTCTCGGCAGTGACTTT |
| **NXK2-5** | TTTGCATTCACTCCTGCGGAGACCTA | ACTCATTGCACGCTGCATAATCGC |
| **MYL2v** | TGTCCCTACCTTGTCTGTTAGCCA | ATTGGAACATGGCCTCTGGATGGA |
| **NPPA** | GGGTCTCTGCTGCATTTGTGTCAT | AGAGGCGAGGAAGTCACCATCAAA |
| **KCNJ3** | TCATCAAGATGTCCCAGCCCAAGA | CACCCGGAACATAAGCGTGAGTTT |
| **HCN4** | TCTTCCTCATTGTGGAGACACGCA | TGAGGATCTTCGTGAAGCGGACAA |
| **SHOX2** | AAGAGGATGCGAAAGGGATG | TGAGTTGTTCCAGGGTGAAAT |

**Supplementary Table 7. Single cell RNA sequencing hashtag barcode sequences**

| Sample batch | Antibody | Dilution | Sequence | Cat. No. |
|---|---|---|---|---|
| **Sample 29** | Anti-human Hashtag 1 Antibody | 1:100 | GTCAACTCTTTAGCG | 394601 |
| **Sample 32** | Anti-human Hashtag 2 Antibody | 1:100 | TGATGGCCTATTGGG | 394603 |
| **Sample 65** | Anti-human Hashtag 3 Antibody | 1:100 | TTCCGCCTCTCTTTG | 394605 |
| **Sample 66** | Anti-human Hashtag 4 Antibody | 1:100 | AGTAAGTTCAGCGTA | 394607 |
| **Sample 67** | Anti-human Hashtag 5 Antibody | 1:100 | AAGTATCGTTTCGCA | 394609 |
| **Sample 68** | Anti-human Hashtag 6 Antibody | 1:100 | GGTTGCCAGATGTCA | 394611 |
| **Sample 70** | Anti-human Hashtag 7 Antibody | 1:100 | TGTCTTTCCTGCCAG | 394613 |
| **Sample 71** | Anti-human Hashtag 8 Antibody | 1:100 | CTCCTCTGCAATTAC | 394615 |
| **Sample 73** | Anti-human Hashtag 9 Antibody | 1:100 | CAGTAGTCACGGTCA | 394617 |
| **Sample 79** | Anti-human Hashtag 10 Antibody | 1:100 | ATTGACCCGCGTTAG | 394619 |
| **Sample 88/90** | Anti-human Hashtag 12 Antibody | 1:100 | TAACGACCAGCCATA | 394623 |
| **Sample 86RA** | Anti-human Hashtag 14 Antibody | 1:100 | CTGTATGTCCGATTG | 394627 |
| **Sample 88RA** | Anti-human Hashtag 15 Antibody | 1:100 | TAAGATTCAGAGCGA | 394629 |

**Supplementary Table 8. Antibodies used for immunohistochemistry experiments.**

| Company | Product | Marker (Primary)<br>Fluorophore (Secondary) | Dilution | Antigen Retrieval (Primary)<br>Host & Target (Secondary) |
|---|---|---|---|---|
| | | *Primary Antibodies* | | |
| **Dako** | M0851 | Alpha smooth muscle actin | 1:200 | Sodium citrate pH6 |
| **DSHB** | CT3 | Cardiac Troponin T | 2μg/mL | Sodium citrate pH6 |
| **Invitrogen** | PA5-47374 | CD200 | 1:20 | Sodium citrate pH6 |
| **Merck Millipore** | AB1728 | Connexin 43 | 1:100 | Sodium citrate pH6 |
| **Cell Signalling Technology** | C48E7 | Ku80 | 1:500 | Sodium citrate pH6 |
| | 91144S | Collagen, type I, alpha 1 | 1:200 | Sodium citrate pH6 |
| **Synaptic Systems** | 311011 | MLC-2a | 1:200 | Sodium citrate pH6 |
| **Abcam** | ab79935 | MLC-2v | 1:100 | Sodium citrate pH6 |
| | ab260039 | SIRPα | 1:100 | Sodium citrate pH6 |
| | ab68167 | Sarcomeric alpha actinin | 1:500 | Sodium citrate pH6 |

| | | | | |
|---|---|---|---|---|
| | ab28364 | CD31 | 1:50 | Sodium citrate pH6 |
| | ab13970 | GFP | 1:150 | Sodium citrate pH6 |
| **Sigma-Aldrich** | C3865 | N-Cadherin | 1:225 | Sodium citrate pH6 |

| | | | | |
|---|---|---|---|---|
| | | *Secondary Antibodies* | | |
| **ThermoFisher** | A-11029 | Alexa Fluor 488 | 1:500 | Goat anti-mouse |
| | A-11034 | Alexa Fluor 488 | 1:500 | Goat anti-rabbit |
| | A-21245 | Alexa Fluor 594 | 1:500 | Goat anti-rabbit |
| | A-21245 | Alexa Fluor 647 | 1:500 | Goat anti-rabbit |
| | A-21236 | Alexa Fluor 647 | 1:500 | Goat anti-mouse |
| **Abcam** | ab150173 | Alexa Fluor 488 | 1:500 | Goat anti-chicken |
| | ab150176 | Alexa Fluor 594 | 1:500 | Goat anti-chicken |
| **Life Technologies** | A11058 | Alexa Fluor 594 | 1:500 | Donkey anti-goat |
